# Comparative Analysis of Acetylated Flavonoids’ Chemopreventive Effects in Different Cancer Cell Lines

**DOI:** 10.3390/ijms25147689

**Published:** 2024-07-13

**Authors:** Daigo Urakawa, Yuki Shioiridani, Shinya Igata, De-Xing Hou, Kozue Sakao

**Affiliations:** 1 The United Graduate School of Agriculture Sciences, Kagoshima University, Kagoshima 890-0065, Japan; k6044078@kadai.jp (D.U.); k8469751@kadai.jp (D.-X.H.); 2 Graduate School of Agriculture, Forestry and Fisheries, Kagoshima University, Kagoshima 890-0065, Japan

**Keywords:** flavonoid, acetylation, modification, anti-cancer, anti-migration, chemoprevention

## Abstract

Flavonoids, a class of natural compounds with anticancer activity, exhibit varying biological activities and potencies based on their structural differences. Acylation, including acetylation of flavonoids, generally increases their structural diversity, which is closely related to the diversity of bioactivity within this group of compounds. However, it remains largely unknown how acetylation affects the bioactivity of many flavonoids. Based on our previous findings that *O*-acetylation enhances quercetin’s bioactivity against various cancer cells, we synthesized 12 acetylated flavonoids, including seven novel compounds, to investigate their anticancer activities in the MDA-MB-231, HCT-116, and HepG2 cell lines. Our results showed that acetylation notably enhanced the cell proliferation inhibitory effect of quercetin and kaempferol across all cancer cell lines tested. Interestingly, while the 5,7,4′-*O*-triacetate apigenin (3Ac-A) did not show an enhanced the effect of inhibition of cell proliferation through acetylation, it exhibited significantly strong anti-migration activity in MDA-MB-231 cells. In contrast, the 7,4′-*O*-diacetate apigenin (2Ac-Q), which lacks acetylation at the 5-position hydroxy group, showed enhanced cell proliferation inhibitory effect but had weaker anti-migration effects compared to 3Ac-A. These results indicated that acetylated flavonoids, especially quercetin, kaempferol, and apigenin derivatives, are promising for anticancer applications, with 3Ac-A potentially having unique anti-migration pathways independent of apoptosis induction. This study highlights the potential application of flavonoids in novel chemopreventive strategies for their anti-cancer activity.

## 1. Introduction

Flavonoids are compounds found in many vegetables and fruits, such as onions, apples, and broccoli [1,2]. Flavonoids are known for their many bioactive compounds, including anticancer, antioxidant, anti-inflammatory, and anti-obesity effects [3,4,5,6]. Also, flavonoids are categorized into the following six groups based on the Flavan skeleton, and according to their structural differences: flavanol, flavone, flavanone, flavonol, isoflavone, and anthocyanidin. It has been reported that the B ring’s catechol structure, the carbonyl group at position 4′, the double bond at C2–C3, and the hydroxyl group at position 3 play an important role in these bioactivities [7]. Furthermore, the diversity of secondary metabolites, including flavonoid subclasses, results from structural modifications such as hydroxylation, methylation, acylation, and glycosylation of the basic skeleton. These modifications, in turn, affect the physicochemical properties, stability, solubility, and bioavailability of the compounds [8,9,10]. The bioactivity of flavonoids is, thus, strongly related to their chemical structure.

One of the biological activities of flavonoids that has been extensively studied is their anticancer effect. Many types of flavonoids exhibit anticancer effects by inhibiting cancer cell proliferation and metastasis, inhibiting angiogenesis, or inducing apoptosis [11,12,13,14]. It has also been shown that a diet rich in flavonoids, which are epigenetic modifiers, is effective in preventing the development and progression of cancer cells [15,16]. Additionally, a report indicates an inverse correlation between flavonoid intake and mortality in patients diagnosed with invasive breast cancer [17]. However, the effective utilization of these flavonoids is hindered by major obstacles such as low membrane permeability, limited systemic bioavailability, rapid metabolic conversion, and oxidative degradation [18]. To overcome these challenges, scientific modifications of flavonoids have been investigated [19]. The aforementioned methylation, glycosylation, and acylation of flavonoids have been well studied as means of scientific modification, not only in plants.

Flavonoid glycosylation modulates the physiological activity of modified forms by altering the solubility and stability of the molecules. Glycosylated compounds can increase drug solubility by more than twofold, enhancing uptake in vitro [20]. However, while glycosylated genistein exhibits higher solubility than its parent compound, it shows no significant improvement in anticancer activity [21], indicating that glycosylation alone may be insufficient to enhance biological activity. Additionally, the direct glycosylation processes of some flavonoids can result in lower yields due to decomposition during synthesis [22], presenting challenges for scientific modification.

Flavonoid methylation is widely observed in nature. In the case of quercetin, for example, methylated derivatives ranging from mono-*O*-methylated to penta-*O*-methylated quercetin have been identified, and their biological activities have been evaluated [23]. Methylated flavonoids generally exhibit higher metabolic stability, oral bioavailability, and biological activity compared to their unmethylated counterparts [10,24]. This suggests that methylation is a beneficial modification for enhancing the efficacy of flavonoids. Research on methylated flavonoids extends beyond major compounds like quercetin [25] and apigenin to include less common derivatives such as tricetin [26]. Many studies have investigated their structure–activity relationships, providing valuable insights into how the position and number of methyl groups can enhance or diminish their biological activity [27,28].

In plants, acylation predominantly occurs as either aromatic or aliphatic acylation, often modifying sugar residues [29,30]. This modification is important for enhancing molecular stability, improving water solubility, and increasing resistance to enzymatic degradation [31,32]. Acetylation has also been identified as a modification of sugar residues, contributing to the cytoprotective effects [33] and anti-inflammatory effects in vivo [34]. On the other hand, while flavonoids with direct modification of the hydroxy group, such as *O*-glycosylation or *O*-methylation, are common in plants, *O*-acylated flavonoids are less frequently observed. Therefore, it is necessary and valuable to obtain *O*-acylated flavonoids through synthesis [35]. The *O*-acylation of polyphenols via enzyme-catalyzed and chemical methods can improve their stability, reduce degradation, increase membrane permeability, and enhance their bioactivity, such as inducing apoptosis in cancer cells [36,37,38,39]. These methods allow the use of a wide range of acyl donors and offer numerous benefits that are not achievable through natural means. The acetyl group, the smallest acyl donor, can easily and effectively modify hydroxy groups in flavonoids like methyl groups, often enhancing their biological activity. For example, acetylating just a single hydroxy group on the B ring of myricetin reduced its oxidation rate by approximately 3.1-fold [40]. Our previous studies also have shown that acetylating quercetin improves its uptake and metabolic stability in HepG2 cells, enhancing apoptosis induction compared to unmodified quercetin [41]. Furthermore, acetylated quercetin exhibited stronger apoptotic effects in human leukemia HL-60 cells and MCF-7 and MDA-MB-231 breast cancer cells by inhibiting proliferation, activating caspase-3 and PARP, and inducing DNA fragmentation. In contrast, methylated quercetin showed a reduced apoptosis-inducing effect in these experiments, suggesting that acetylation may, in certain contexts, offer comparative advantages over methylation for enhancing apoptotic activity [23,42]. Similar anticancer-promoting effects have been reported for acetylated chrysin and luteolin [43,44]. However, despite the potential of acetylated flavonoids to offer benefits comparable to methylated flavonoids, research on acetylated derivatives of other flavonoids remains limited. This has created a notable gap in understanding the structure-activity relationships of acetylated flavonoids, especially when compared to the extensive studies conducted on methylated flavonoids. As research in acylation advances, exploring the potential benefits of acetylated flavonoids may reveal advantages comparable to or even surpassing those of methylated counterparts. In this study, based on previous studies on acetyl derivatives of quercetin, eight flavonoids were selected from flavonols, flavones, and flavanones, focusing on the structure of quercetin. We synthesized acetylated derivatives, comprising seven novel compounds, and conducted a comparative analysis of their chemopreventive effects in different cancer cell lines.

In 2022, the second and third leading causes of cancer-related deaths in men were colorectal cancer (9.3% of total cancer deaths) and liver cancer (7.8% of total cancer deaths), respectively. For women, the leading cause was breast cancer (6.9% of total cancer deaths), followed by colorectal cancer (7.8% of total cancer deaths) [45]. Quercetin has been extensively studied for its chemopreventive properties against these three cancers [46]. Furthermore, our findings have already shown that *O*-acetylation of quercetin enhances its anticancer activity against liver cancer (HepG2) and breast cancer (MDA-MB-231 and MCF-7). Therefore, to extensively investigate the effects of acetyl modification, we tested its ability to inhibit cell proliferation in MDA-MB-231 (breast cancer), HCT-116 (colon cancer), and HepG2 (liver cancer) cells, and examined the structure–activity relationships that enhance physiological activity. Furthermore, we have identified the effect of flavonoid acetylation on the inhibition of cancer cell migration. MDA-MB-231 cells are well-known for their high metastatic potential in breast cancer. Our previous studies showed that acetylated quercetin induced higher apoptotic cell death in MDA-MB-231 cells compared to unmodified quercetin. However, its effect on metastasis inhibition remains unclear. Several reports have investigated the anti-metastatic potential of flavonoids [47]. The eight flavonoids used in this study—kaempferol [48], quercetin [49], myricetin [50], luteolin [51], chrysin [52], apigenin [53], Naringenin [54], and Taxifolin [55]—have demonstrated metastasis inhibitory effects in breast cancer cells. Therefore, elucidating the impact of acetylation on these metastasis inhibitory effects will provide new insights into the chemopreventive potential of flavonoids.

To the best of our knowledge, this is the first study to investigate the impact of acetylation on the anticancer and antimigration activities of multiple flavonoids, with a focus on structure–activity relationships. Additionally, our evaluation of apigenin revealed that its acetylation can confer unique anti-migratory properties distinct from other flavonoids. This finding highlights the potential of acetylated flavonoids in developing new strategies for chemical prevention, particularly in the context of anticancer effects.

## 2. Results

### 2.1. Synthesis and Structural Characterization of Acetyl Flavonoids

**Figure 1:**
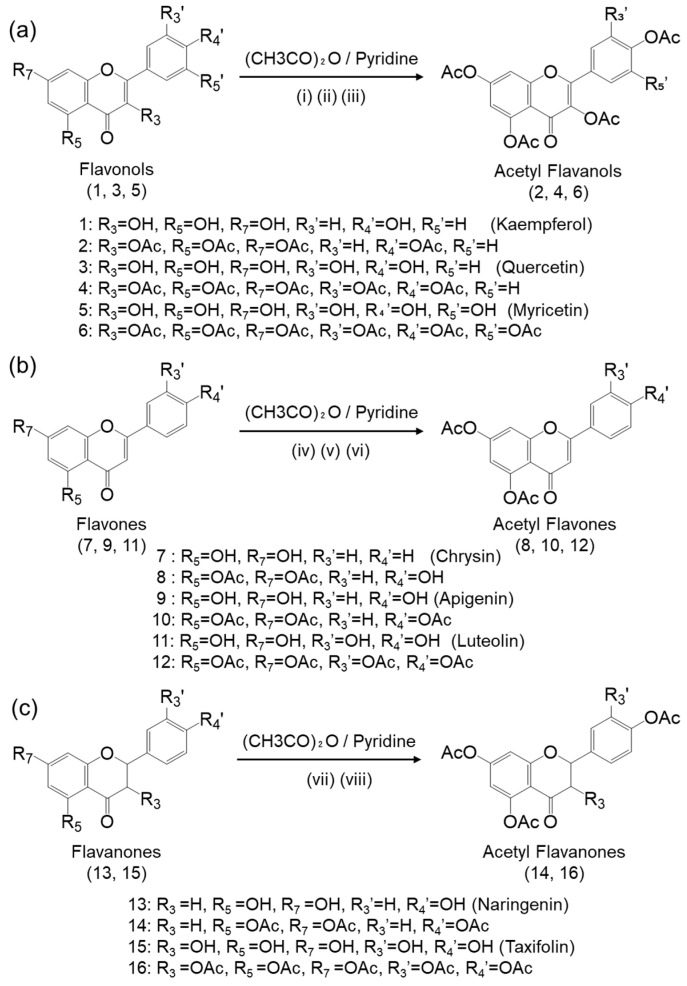
Scheme for the acetylation of flavonols, flavones, and flavanones. (**a**) The conditions for the acetylation of flavonols were as follows: for compound **2**, (i) (CH_3_CO)_2_O (5 eq.), 25 °C, 12 h; for compound **4**, (ii) (CH_3_CO)_2_O (6 eq.), 25 °C, 2 h; and for compound **6**, (iii) (CH_3_CO)_2_O (7 eq.), 25 °C, 12 h. (**b**) The conditions for the acetylation of flavones were as follows: for compound **8**, (iv) (CH_3_CO)_2_O (3 eq.), reflux, 7 h; for compound **10**, (v) (CH_3_CO)_2_O (4 eq.), reflux, 7 h; and for compound **12**, (vi) (CH_3_CO)_2_O (5 eq.), reflux, 12 h. (**c**) The conditions for the acetylation of flavanones were as follows: for compound **14**, (vii) (CH_3_CO)_2_O (100 eq.), 25 °C, 5 h; for compound **16**, (viii) (CH_3_CO)_2_O (8 eq.), 37 °C, 1 h.

Acetyl-flavonols (compounds **2**, **4**, and **6**), acetyl-flavones (compounds **8**, **10**, **12**, and **20**), and acetyl-flavanones (compounds **14** and **16**) were synthesized by reacting the parent flavonoids with acetic anhydride in the presence of pyridine as a catalyst (Figure 1). The products were purified by recrystallization. As described below, the chemical structures were determined by ^1^H-NMR and FT-IR analyses.

Successful acetylation was confirmed by the disappearance of the hydroxyl group signals in the ^1^H-NMR spectra of the parent flavonoids (Table S1) and/or the appearance of new signals around 2.3 ppm corresponding to the acetyl groups in the spectra of the acetylated derivatives. Additionally, the integral ratios for each compound were consistent with the expected structures. In the FT-IR spectra, acetylation was evidenced by the disappearance of the hydroxyl group absorption band (3000–3500 cm^−1^) present in the parent flavonoids and the appearance of a characteristic carbonyl absorption band around 1700 cm^−1^ from the acetyl groups. Additionally, the C-O stretching vibrations of the acetyl groups were also observed in the region of 1100–1300 cm^−1^. These spectral changes clearly indicate that the hydroxyl groups of the flavonoids were acetylated. Detailed spectral data for each acetyl-flavonoid are provided, including the compound name, physical state, yield, melting point (m.p.), ^1^H-NMR spectrum, and FT-IR spectrum, as shown below.

Compound **2**: 3,5,7,4′-*O*-tetraacetate kaempferol (4Ac-K), White powder; Yield: 71.0 ± 2.12 (%); m.p. 126 °C; ^1^H-NMR (600 MHz, DMSO-D_6_) δ: 7.96 (2H, d, *J* = 8.7 Hz), 7.65 (1H, d, *J* = 2.1 Hz), 7.39 (2H, d, *J* = 8.7 Hz), 7.18 (1H, d, *J* = 2.1 Hz), 2.33 (12H, dd, *J* = 10.6, 1.3 Hz). FT-IR (cm^−1^): 1761 (C=O), 1154 (C-O). A ^1^H-NMR signal derived from an acetyl group was observed at 2.33 ppm, with an integration ratio of 12H, suggesting the presence of four *O*-acetyl.

Compound **4**: 3,5,7,3′,4-*O*-pentaacetate quercetin (5Ac-Q), white powder; yield: 70.8 ± 11.7 (%); m.p. 226 °C; ^1^H-NMR (600 MHz, CDCl_3_) δ: 7.71 (2H, dt, *J* = 16.9, 6.1 Hz), 7.35 (1H, dd, *J* = 10.3, 5.3 Hz), 6.88 (1H, d, *J* = 2.1 Hz), 2.35 (15H, dd, *J* = 3.3, 1.2 Hz). FT-IR (cm^−1^): 1760 (C=O), 1175 (C-O). A ^1^H-NMR signal derived from an acetyl group was observed at 2.35 ppm, with an integration ratio of 15H, suggesting the presence of five *O*-acetyl.

Compound **6**: 3,5,7,3′,4′,5′-*O*-hexaacetate myricetin (6Ac-M), white powder; yield: 67.2 ± 10.7 (%); m.p. 231 °C; ^1^H-NMR (600 MHz, 600 MHz, DMSO-D_6_) δ: 7.80 (2H, d, *J* = 0.7 Hz), 7.66 (1H, dd, *J* = 2.2, 0.7 Hz), 7.18 (1H, dd, *J* = 2.2, 0.7 Hz), 2.34 (18H, s). FT-IR (cm^−1^): 1765 (C=O), 1131 (C-O). A ^1^H-NMR signal derived from an acetyl group was observed at 2.34 ppm, with an integration ratio of 18H, suggesting the presence of six *O*-acetyl.

Compound **8**: 5,7-*O*-diacetate chrysin (2Ac-C), pale-white powder; yield: 57.7 ± 2.38 (%); m.p. 190 °C; ^1^H-NMR (600 MHz, DMSO-D_6_) δ: 8.09 (2H, t, *J* = 4.2 Hz), 7.64 (1H, dd, *J* = 2.2, 0.7 Hz), 7.60 (2H, d, *J* = 7.9 Hz), 7.17–7.09 (2H, m), 6.94 (1H, s), 2.34 (6H, d, *J* = 4.2 Hz). FT-IR (cm^−1^): 1766 (C=O), 1182 (C-O). A ^1^H-NMR signal derived from an acetyl group was observed at 2.34 ppm, with an integration ratio of 6H, suggesting the presence of two *O*-acetyl.

Compound **10**: 5,7,4′-*O*-triacetate apigenin (3Ac-A), brown powder; yield: 55.2 ± 1.97 (%); m.p. 197 °C; ^1^H-NMR (600 MHz, CDCl_3_) δ: 7.47 (2H, d, *J* = 8.6 Hz), 7.16 (2H, t, *J* = 4.3 Hz), 6.79–6.78 (1H, m), 6.55 (1H, dd, *J* = 2.5, 1.4 Hz), 5.50 (1H, dd, *J* = 13.7, 2.5 Hz), 3.04 (1H, dd, *J* = 16.7, 13.7 Hz), 2.78 (1H, dd, *J* = 16.7, 2.7 Hz), 2.35 (9H, dd, *J* = 44.9, 6.3 Hz). FT-IR (cm^−1^): 1751 (C=O), 1188 (C-O). A ^1^H-NMR signal derived from an acetyl group was observed at 2.35 ppm, with an integration ratio of 9H, suggesting the presence of three *O*-acetyl.

Compound **12**: 5,7,3′,4′-*O*-tetraacetate luteolin (4Ac-L), white powder; yield: 71 ± 4.24 (%) %; m.p. 214 °C; ^1^H-NMR (600 MHz, CDCl_3_) δ: 7.78 (2H, dd, *J* = 8.5, 2.1 Hz), 7.74 (2H, d, *J* = 2.2 Hz), 7.39 (1H, d, *J* = 8.5 Hz), 6.86 (1H, d, *J* = 2.0 Hz), 2.36–2.34 (12H, m). FT-IR (cm^−1^): 1764 (C=O), 1185 (C-O). A ^1^H-NMR signal derived from an acetyl group was observed at 2.34–2.36 ppm, with an integration ratio of 12H, suggesting the presence of four *O*-acetyl.

Compound **14**: 5,7,4′-*O*-triacetate naringenin (3Ac-N), white powder; yield: 25.8 ± 6.22 (%); m.p. 127 °C; ^1^H-NMR (600 MHz, CDCl_3_) δ: 7.47 (2H, d, *J* = 8.6 Hz), 7.16 (2H, d, *J* = 6.6 Hz), 6.79–6.78 (1H, m), 6.54 (1H, t, *J* = 1.1 Hz), 5.50 (1H, dd, *J* = 13.7, 2.5 Hz), 3.06 (1H, s), 3.04 (1H, d, *J* = 3.1 Hz), 3.01 (1H, s), 2.39 (9H, s). FT-IR (cm^−1^): 1748 (C=O), 1169 (C-O). A ^1^H-NMR signal derived from an acetyl group was observed at 2.39 ppm, with an integration ratio of 9H, suggesting the presence of three *O*-acetyl.

Compound **16**: 3,5,7,3′,4′-*O*-pentaacetate taxifolin (5Ac-T), white powder; yield: 72.0 ± 11.3 (%); m.p. 138 °C; ^1^H-NMR (600 MHz, CDCl_3_) δ: 7.39 (1H, t, *J* = 4.4 Hz), 7.29 (2H, d, *J* = 2.1 Hz), 6.79 (1H, t, *J* = 1.1 Hz), 6.61 (1H, t, *J* = 1.1 Hz), 5.66 (1H, d, *J* = 12.3 Hz), 5.43 (1H, d, *J* = 12.3 Hz), 2.34 (15H, d, *J* = 26.7 Hz). FT-IR (cm^−1^): 1760 (C=O), 1177 (C-O). A ^1^H-NMR signal derived from an acetyl group was observed at 2.34 ppm, with an integration ratio of 15H, suggesting the presence of five *O*-acetyl.

### 2.2. Acetylated Flavonoids Inhibit the Proliferation of Three Types of Cancer

Many previously reported acetylated flavonoids, including those in our study, have demonstrated increased inhibition of cell proliferation compared to their parent compounds. In this study, we assessed the cell proliferation inhibitory effects of acetylated forms of various flavonoids on the MDA-MB-231, HCT-116, and HepG2 cell lines. We determined the 50% Inhibition Concentration (IC_50_) after 48 h of treatment, with acetylated quercetin showing the most pronounced inhibitory effect on cell proliferation in our previous investigation [23,41]. The IC_50_ values of each parent compound and its acetylated derivatives for these three cell types were summarized in Table 1.

First, the cell proliferation inhibition effect of each compound on MDA-MB-231 cells was measured using the trypan blue exclusion assay. As Table 1 shows, the inhibitory concentrations (IC_50_) of the original compounds, flavonols such as kaempferol, quercetin, and myricetin, were 46.7 µM, 24.3 µM, and 27.2 µM, respectively. The IC_50_ values of their acetylated derivatives, 4Ac-K, 5Ac-Q, and 6Ac-M, were 33.6 µM, 17.4 µM, and 50.9 µM, respectively. These results indicate that acetylation enhanced the tumor cell growth inhibitory potential of kaempferol and quercetin by approximately 1.39-fold and 1.40-fold, respectively. In contrast, the acetylation of myricetin resulted in a decreased inhibitory effect, with the IC_50_ value increasing by 1.87-fold. For flavones such as chrysin, apigenin, and luteolin, the IC_50_ values were 37.5 µM, 27.1 µM, and 12.9 µM, respectively. Their acetylated derivatives, 2Ac-C, 3Ac-A, and 4Ac-L had IC_50_ values of 38.4 µM, 31.1 µM, and 20.2 µM, respectively. These data suggest that acetylation did not significantly affect the ability of chrysin and apigenin to inhibit tumor cell proliferation, with changes in IC_50_ values being minimal (1.02-fold and 1.15-fold increase, respectively). However, the acetylation of luteolin decreased its inhibitory effect, increasing the IC_50_ value by 1.57-fold. Flavanones such as naringenin and taxifolin had IC_50_ values above 160 µM. The IC_50_ values of their acetylated derivatives, 3Ac-N and 5Ac-T, were 156.4 µM and 128.0 µM, respectively. The IC_50_ values for both naringenin and taxifolin exceeded 160 µM, and acetylation slightly improved their inhibitory effect, reducing the IC_50_ values to 156.4 µM (1.02-fold) and 128.0 µM (1.25-fold), respectively. This suggests that acetylation may have enhanced the inhibitory effect on cell proliferation for these flavanones, although the changes were not substantial. Overall, these results demonstrate that acetylation can either enhance or reduce the anticancer activity of flavonoids depending on the specific compound. Kaempferol and quercetin derivatives showed significant improvements, while myricetin and luteolin derivatives exhibited a decreased effectiveness. The impact of acetylation on flavones and flavanones was less pronounced but still indicated the potential modulation of bioactivity.

The cell growth inhibitory effects of each flavonoid and its acetylated derivatives on HCT-116 cells were subsequently determined in a similar manner. The IC_50_ values of kaempferol and quercetin were 34.85 µM and 23.45 µM, respectively, while that of myricetin exceeded 160 µM. The IC_50_ values of their acetylated derivatives, 4Ac-K, 5Ac-Q, and 6Ac-M, were 28.53 µM, 15.66 µM, and 81.66 µM, respectively. These results suggest that acetylation enhanced the tumor cell proliferation inhibitory ability of kaempferol and quercetin by approximately 1.22-fold and 1.50-fold, respectively. In contrast, acetylation of myricetin reduced the IC_50_ value to 81.66 µM, which was about twice as strong as the parent compound. However, it should be noted that the IC_50_ value of the parent compound is above 160 µM, indicating it was not highly potent to begin with. The IC_50_ values of the chrysin, apigenin, and luteolin were 27.5 µM, 19.0 µM, and 9.3 µM, respectively. Upon acetylation, their inhibitory potency was moderately affected, with IC_50_ values of 32.2 µM for 2Ac-C, 21.9 µM for 3Ac-A, and 12.2 µM for 4Ac-L. These results suggest that acetylation led to slight increases in IC_50_ values by factors of approximately 1.17, 1.15, and 1.31, respectively, indicating a modest reduction in inhibitory effect due to acetylation. The IC_50_ of the naringenin was 120.4 µM, and that of taxifolin exceeded 160 µM. The IC_50_ values of 3Ac-N and 5Ac-T, were 94.3 µM and 125.8 µM, respectively. These results suggest that acetylation of taxifolin reduces its IC_50_ value by at least about 1.27-fold and increases its ability to inhibit tumor cell proliferation. The ability of naringenin to inhibit HCT-116 cell proliferation was also increased by acetylation, with an approximately 1.28-fold decrease in IC_50_ value. Similar to the evaluation using MDA-MB-231 cells, acetylation demonstrated improvements in cell proliferation inhibition for kaempferol and quercetin, with a slight decrease observed for flavones and an enhancement for flavanones. Myricetin showed at least a 2-fold increase in activity.

Furthermore, the inhibitory effect on cell proliferation in HepG2 cells was also measured using the same method. Kaempferol and quercetin exhibited IC_50_ values of 33.38 µM and 28.16 µM, respectively, with myricetin exceeding 160 µM. Acetylated derivatives 4Ac-K, 5Ac-Q, and 6Ac-M showed IC_50_ values of 23.2 µM, 15.5 µM, and 76.6 µM, respectively, indicating an approximately 1.4-fold, 1.8-fold, and 2.1-fold enhancement in inhibitory activity compared to their respective parent compounds. For flavones, chrysin, apigenin, and luteolin demonstrated IC_50_ values of 25.3 µM, 25.8 µM, and 10.2 µM, respectively. The IC_50_ values of 2Ac-C, 3Ac-A, and 4-Ac-L were 23.8 µM, 6.5 µM, and 12.4 µM, respectively. These results indicate that the acetylation of apigenin enhances its tumor cell proliferation inhibitory activity by approximately 4-fold, while the acetylation of luteolin decreases its inhibitory activity by about 1.2-fold. Chrysin showed a slight improvement in its inhibitory effect with an IC_50_ reduction of about 1.1-fold. The flavone naringenin showed an IC_50_ of 120.4 μM and taxifolin exceeded 160 μM. The acetylated derivatives, 3Ac-N and 5Ac-T, showed IC_50_ values of 97.9 μM and 152.2 μM, respectively, suggesting an approximately 1.2-fold and at least 1.1-fold increase in inhibitory activity over the parent compound, respectively.

### 2.3. Effects of Flavonoid Acetylation on Cell Cycle Progression-Related Proteins

Based on the demonstrated enhancement of cell proliferation inhibitory ability through acetylation in all three cell types by kaempferol and quercetin, experiments were conducted to investigate the relationship between cell proliferation inhibitory ability and the cell cycle-related proteins. In cellular experiments aimed at identifying cell cycle stages and the movement of cell cycle-related proteins, accurately discerning the behavior of cells in different stages when mixed poses a challenge. Therefore, Western blotting was conducted using cells synchronized in the S phase with hydroxyurea (HU) in the present experiment. Synchronization via HU treatment induces cell arrest in the late G1/early S phase, and subsequent release from HU-mediated arrest yields a cell population that uniformly progresses through S and G2/M phases.

**Figure 2:**
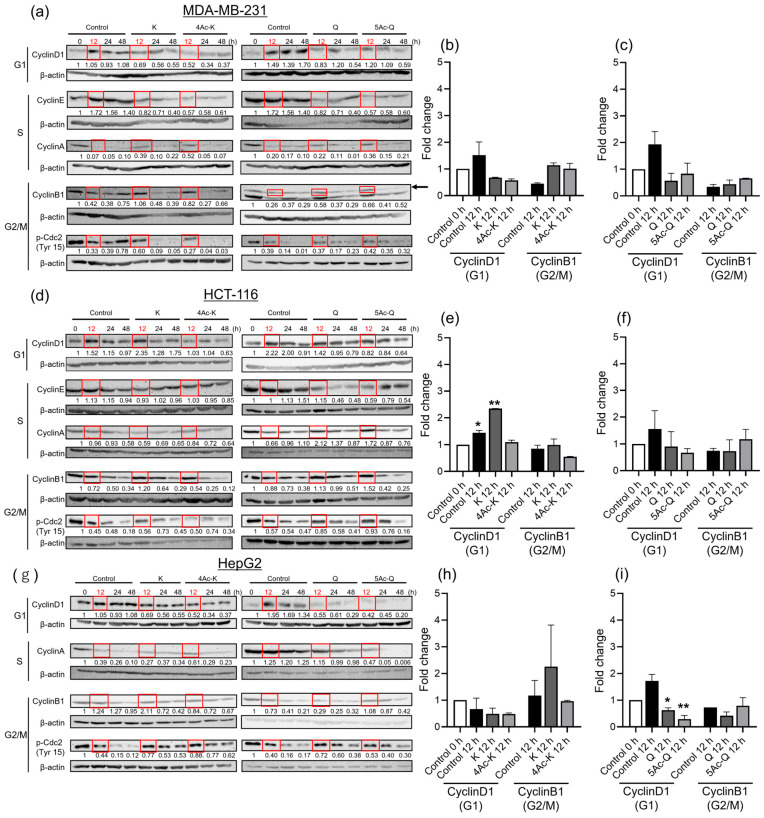
Western blot of cytoplasmic signaling proteins related to cell cycle progression. Results are shown for checkpoint promoters for G1-phase progression (CyclinD1) and regulators for S-phase progression (CyclinA/E); regulators for G2/M transition (CyclinB1 and p-Cdc2) in MDA-MB-231 (**a**), HCT-116 (**d**), and HepG2 (**g**) cell lines. Blots were stripped and re-probed with anti-actin antibody to correct for differences in protein loading. Numbers on bottom of bands are fold changes in expression level relative to corresponding DMSO-treated control. Red square indicate the 12 h blots in which the cycle was arrested. Quantitative data for protein expression of kaempferol and 4Ac-K (**b**,**e**,**h**) and quercetin and 5Ac-Q (**c**,**f**,**i**) in each cell, normalized by β-actin expression. Each value in the graph represents the mean ± SD (*n* = 2–4) of repeated experiments. * *p* < 0.05, ** *p* < 0.01, indicating a significant difference between the control and each sample. For Western blot analysis, 1 mM hydroxyurea was added 24 h prior to sample addition and all cell cycles were synchronized in S phase. Cells were treated with 40 μM of kaempferol (K) or 4Ac-K, and quercetin (Q) or 5Ac-Q for indicated time, then the harvested cell lysate was used. Western blotting was performed two to four times using independently prepared lysates.

Each compound’s cytoplasmic signaling proteins associated with cell cycle progression relative to MDA-MB-231 cells were detected by Western blotting. As shown in Figure 2a, kaempferol and its acetylated derivative 4Ac-K showed the upregulation of CyclinA (S phase) and CyclinB1 (G2/M phase) at 12 h compared to the control group at the same time point. Compared to the 0 h baseline, the fold changes of CyclinA and Cyclin B1 in the control group decreased to 0.07 and 0.42, respectively, at 12 h. In contrast, treatment with kaempferol for 12 h showed fold changes of 0.39 and 1.06, respectively. Similarly, treatment with 4Ac-K resulted in fold changes of 0.52 for cyclin A and 0.82 for cyclin B1.

Setting the 12 h control value as 1, the fold change in CyclinA with kaempferol treatment was 5.57, and with 4Ac-K treatment it was 7.43. For Cyclin B1, the fold change with kaempferol treatment was 2.52, and with 4Ac-K treatment it was 1.95. The checkpoint control factor for G1 transition, CyclinD1, was downregulated to approximately half of the control level at the 12 h point with both kaempferol and 4Ac-K (Figure 2b). Additionally, although it was not statistically significant between the samples, both showed an increase in p-Cdc2 compared to the control. Quercetin reduced the expression of CyclinD1and CyclinE, and slightly increased CyclinB1 at 12 h, while 5Ac-Q decreased CyclinE to more than one third of the control and slightly increased CyclinA, CyclinB1, and p-Cdc2 (Figure 2a,c). These findings suggested that Kaempferol, 4Ac-K, quercetin, and 5Ac-Q may regulate cell cycle-related proteins associated with G1 or G2/M phases.

In HCT-116 cells, as shown in Figure 2d, treatment with kaempferol for 12 h reduced CyclinE and CyclinA expression, while increasing CyclinD1 levels by 1.5-fold and CyclinB1 levels by 1.7-fold (Figure 2e). Additionally, phosphorylated-Cdc2 (p-Cdc2) levels increased by 1.2-fold. Subsequently, CyclinD1 and CyclinB1 decreased over time in a time-dependent manner. Conversely, 4Ac-K did not significantly alter CyclinE and CyclinA expression compared to the controls, but it exhibited lower fold changes for CyclinD1 (0.67-fold) and CyclinB1 (0.75-fold) compared to the controls. The CyclinD1 and CyclinB1 levels continued to decrease over time, suggesting a G2/M phase arrest induced by 4Ac-K. These results suggest that kaempferol and 4Ac-K may influence different cell cycle-related proteins.

After 12 h of treatment, quercetin and 5Ac-Q exhibited similar effects on checkpoint-related proteins (Figure 2d,f). Both compounds significantly decreased CyclinD1 levels (0.63-fold for quercetin and 0.37-fold for 5Ac-Q) and increased CyclinA (3.2-fold for quercetin and 2.6-fold for 5Ac-Q), CyclinB1 (1.3-fold for quercetin and 1.7-fold for 5Ac-Q), and p-Cdc2 (1.5-fold for quercetin and 1.6-fold for 5Ac-Q). Subsequently, CyclinD1, CyclinB1, and p-Cdc2 levels decreased over time, suggesting that Cdc2 inactivation was accompanied by an increased accumulation of CyclinB1 in the cytosol. These findings indicate that proteins associated with the G2/M phase were similarly affected by both quercetin and 5Ac-Q treatments.

In HepG2 cells (Figure 2g), both kaempferol and 4Ac-K showed decreased CyclinD1 levels (0.65-fold and 0.49-fold, respectively) compared to the controls after 12 h. Kaempferol increased CyclinB1 levels by 1.7-fold and p-Cdc2 levels by 1.8-fold, while 4Ac-K exhibited fold changes of 0.7-fold for CyclinB1 and 2.0-fold for p-Cdc2. The fold changes in CyclinD1 and p-Cdc2 proteins were greater for 4Ac-K compared to kaempferol. In contrast, both quercetin and 5Ac-Q decreased CyclinD1 levels to nearly a quarter of the control. Quercetin showed an increase in p-Cdc2 levels by 1.8-fold, with no significant change in CyclinB1 levels over 12–24 h. On the other hand, 5Ac-Q exhibited an increase in CyclinB1 levels by 1.5-fold and p-Cdc2 levels by 1.3-fold at 12 h, followed by a decrease. These findings suggest that in HepG2 cells, acetyl derivatives affect cell cycle-associated proteins in the G2/M phase similar to the parent and child products.

### 2.4. Screening of Acetylated Flavonoids for Anti-Migration Properties

**Figure 3:**
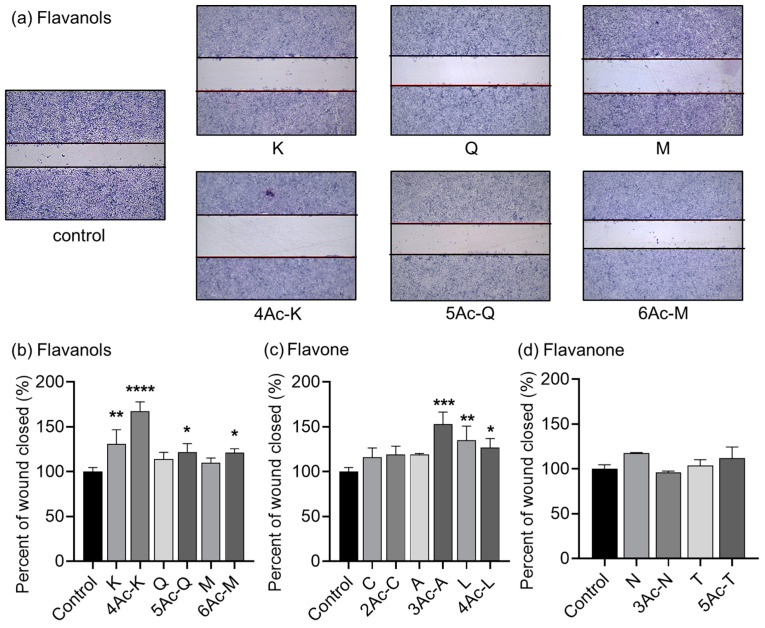
Assessment of anti-cellular migration using wound healing assay. (**a**) Representative images of scratch assay showing the effect of flavonols (K: kaempferol, Q: quercetin, M: myricetin) and acetyl flavonols treatments (6 h treatment) on wound healing in MDA-MB-231 cells. (**b**–**d**), the wound healing quantification of flavonols, flavones (C: chrysin, A: apigenin, L: luteolin), flavanones (N: naringenin, T: taxifolin) and their acetyl derivatives. The results shown (mean ± SD) are representative of two independent experiments (*n* = 9). * mark denoted significant differences (* *p* < 0.05, ** *p* < 0.01, *** *p* < 0.001, **** *p* < 0.0001) between the control and each compound using one-way ANOVA followed by Dunnett’s multiple comparison test.

We evaluated whether acetylation of flavonoids also affects the anti-migration potential of MDA-MB-231 cells. In the wound healing assay, we set the sample treatment time to 6 h since wounds heal within 9 h. Additionally, to observe a significant difference, we treated the cells at twice the IC_50_ concentration obtained from the 48 h cell proliferation inhibition assay, as lower concentrations were insufficient to produce measurable effects. The flavonols, all acetylated compounds, showed anti-migration activity. 4Ac-K exhibited the highest anti-migration activity, reducing cell migration to 167.7% compared to the control. Kaempferol and 5Ac-Q reduced cell migration to 131.0% and 122.0%, respectively, while 6Ac-M reduced migration to 121.0% (Figure 3b). For the flavones, 3Ac-A, synthesized from apigenin, demonstrated significantly higher anti-migration activity than the other flavonols, reducing cell migration to 153.1%. Luteolin and 4Ac-L also showed higher anti-migration activity than the control, reducing cell migration to 135.1% and 129.0%, respectively (Figure 3c). Among the flavanones, neither the parent compounds nor their acetylated derivatives exhibited significant migration inhibition. 3Ac-N showed lower anti-migration activity than the original compound, with cell migration at 96.1%. 5Ac-T showed a slight increase in anti-migration activity, reducing migration to 112.0%, though this difference was not statistically significant (Figure 3d). Statistical analysis was performed using a one-way ANOVA followed by Dunnett’s multiple comparison test.

### 2.5. Synthesis of Acetylated Compounds with Retained 5-Hydroxy Group

**Figure 4:**
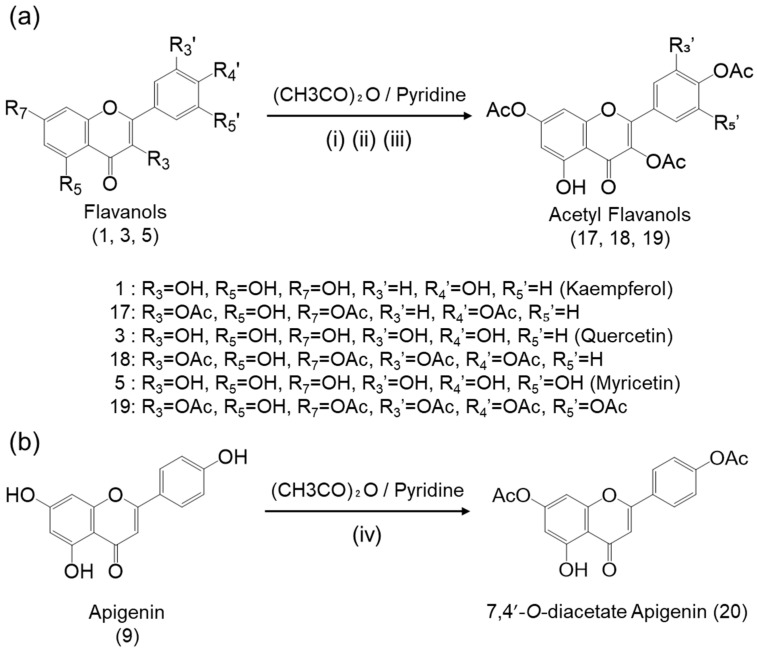
Selective acetylation of hydroxyl groups other than the 5th position hydroxyl group. (**a**) Reagents and condition for compound **17**, (i): (CH_3_CO)_2_O (3.2 eq), 27 °C, 5 min; for compound **18**, (ii): (CH_3_CO)_2_O (4.2 eq), 27 °C, 7 min; for compound **19**, (iii): (CH_3_CO)_2_O (5.2 eq), 27 °C, 12 min; and (**b**) for compound **20**, (iv): (CH_3_CO)_2_O (2 eq), 0 °C, 5 min.

Our previous study reported that among the acetyl derivatives of quercetin, 4Ac-Q, which retained the hydroxy group at position 5 of the A ring, enhanced the ability to induce apoptosis [23]. Therefore, we newly synthesized compounds **17**, **18**, **19**, and **20** (Figure 4), which also retain the hydroxy group at position 5 of the A ring, expecting to further enhance cell proliferation inhibition and antimigration potential.

A hydroxy group at position 5 of the A ring is not easily acetylated due to hydrogen bonding with the carbonyl group at position 4. By shortening the reaction time, controlled acetylation at this position was achieved. Consequently, compounds **17**, **18**, and **19** were successfully synthesized while retaining the hydroxy group at position 5. The ^1^H-NMR results confirmed that the signal around 12 ppm, corresponding to the hydroxy group at position 5 of the A-ring in the parent compound, remained unchanged, while new signals derived from acetyl groups appeared around 2.3 ppm. Furthermore, the integral ratio corresponding to three protons (3H: equivalent to one −COCH_3_ group) was reduced compared to acetylating all hydroxy groups of each parent compound, confirming successful acetylation without modification at the 5 position. Similarly, compound **20** retained the hydroxy group at position 5 of the A ring successfully by adjusting both the temperature and reaction time. The ^1^H-NMR results for compound **20** indicated a decrease in the integral ratio of the protons (3H) corresponding to one acetyl group signal around 2.3 ppm, consistent with the results for compounds **17**, **18**, and **19**. The signal around 13 ppm corresponding to the hydroxy group at position 5 of the A ring of apigenin was outside the measurement range and could not be confirmed. Furthermore, FT-IR analysis of compounds **17**, **18**, **19**, and **20** revealed an additional absorption peak specific to the C=O stretch around 1700 cm^−1^.

Compound **17**: 3,7,4′-*O*-triacetate kaempferol (3Ac-K), pale-yellow powder; yield: 53.0 ± 2.82 (%); m.p. 206°C; ^1^H-NMR (600 MHz, CDCl_3_) δ: 12.15 (1H, s), 7.88 (2H, d, *J* = 8.8 Hz), 7.28 (2H, d, *J* = 8.8 Hz), 6.85 (1H, d, *J* = 2.0 Hz), 6.60 (1H, d, *J* = 2.0 Hz), 2.35 (9H, t, *J* = 5.5 Hz). FT-IR (cm^−1^): 1770 (C=O), 1147 (C-O). A ^1^H-NMR signal derived from an acetyl group was observed at 2.35 ppm, with an integration ratio of 9H, suggesting the presence of three *O*-acetyl groups. Additionally, a peak derived from the hydroxy group at position 5 was observed at 12.15 ppm with an integration ratio of 1H.

Compound **18**: 3,7,3′,4′-*O*-tetraacetate quercetin (4A-Q), pale-yellow powder; yield: 72.1 ± 3.25 (%); m.p. 199 °C; ^1^H-NMR (600 MHz, CDCl_3_) δ: 12.10 (1H, s), 7.75 (2H, dd, *J* = 10.7, 1.8 Hz), 7.37 (1H, d, *J* = 8.3 Hz), 6.86 (1H, d, *J* = 1.6 Hz), 6.61 (1H, d, *J* = 1.6 Hz), 2.36 (12H, dd, *J* = 12.0, 10.2 Hz). FT-IR (cm^−1^): 1764 (C=O), 1204 (C-O). A ^1^H-NMR signal derived from an acetyl group was observed at 2.36 ppm, with an integration ratio of 12H, suggesting the presence of three *O*-acetyl groups. Additionally, a peak derived from the hydroxy group at position 5 was observed at 12.1 ppm with an integration ratio of 1H.

Compound **19**: 3,7,3′,4′,5′-*O*-pentaacetate myricetin (5Ac-M), pale-yellow powder; Yield: 61.0 ± 5.64 (%); m.p.211°C; ^1^H-NMR (CDCl_3_) δ: 12.07 (1H, s), 7.65 (2H, s), 6.86 (1H, d, *J* = 2.0 Hz), 6.61 (1H, d, *J* = 2.0 Hz), 2.37 (15H, dd, *J* = 21.5, 17.6 Hz). 1775 (C=O), 1179 (C-O). A ^1^H-NMR signal derived from an acetyl group was observed at 2.37 ppm, with an integration ratio of 15H, suggesting the presence of three *O*-acetyl groups. Additionally, a peak derived from the hydroxy group at position 5 was observed at 12.07 ppm with an integration ratio of 1H.

Compound **20**: 7,4′-*O*-diacetate apigenin (2Ac-A) brown powder; yield: 67.5 ± 6.57 (%); m.p. 217 °C; ^1^H-NMR (ACETONE-D_6_) δ: 8.20–8.17 (2H, m), 7.52–7.51 (1H, m), 7.39–7.35 (2H, m), 7.03–7.02 (1H, m), 6.98–6.95 (1H, m), 6.62–6.61 (1H, m), 2.84 (5H, tt, *J* = 12.4, 2.3 Hz), 2.33–2.29 (6H, m). FT-IR (cm^−1^): 1753 (C=O), 1189 (C-O). A ^1^H-NMR signal derived from an acetyl group was observed at 2.29–2.33 ppm, with an integration ratio of 6H, suggesting the presence of three *O*-acetyl groups.

### 2.6. Effect of Position 5 Hydroxyl Group on Flavonoids’ Anti-Proliferative Activity

**Figure 5:**
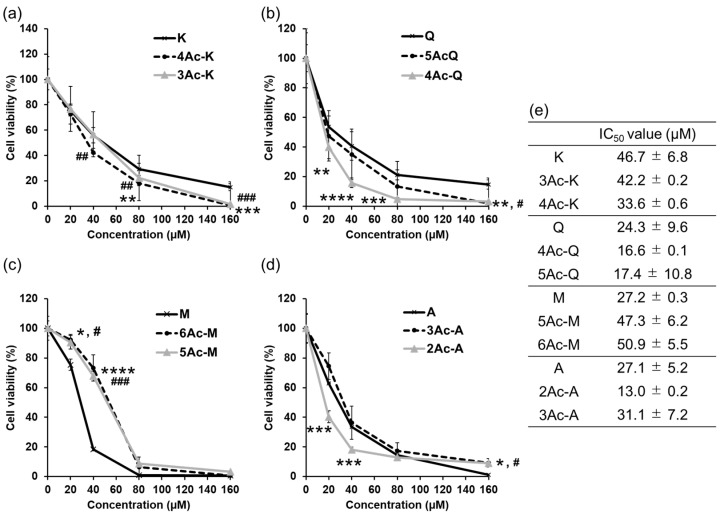
Effect of the existence of the 5-position hydroxyl group of acetylated flavonoids on the proliferation of MDA-MB-231 cells. Cells were treated for 48 h with compounds at concentrations ranging from 0–160 μM or 0.1% DMSO (control). The treated compounds are (**a**) kaempferol (K), (**b**) quercetin (Q), (**c**) myricetin (M), and (**d**) apigenin (A) as parent compounds and their acetyl derivatives, respectively. (**e**) IC_50_ values in μM. IC_50_ values were calculated from dose- − response curves. The data shown represent the means ± SD of three or more independent experiments. *, # *p* < 0.05, **, ## *p* < 0.01, ***, ### *p* < 0.001, **** *p* < 0.0001: significant differences between control and each compound (*: 3Ac-K, 4Ac-Q, 5Ac-Q or 2Ac-A, #: 4Ac-K, 5Ac-Q, 6Ac-Q or 3Ac-A) by Tukey’s multiple range test.

The cell viability of each sample against MDA-MB-231 cells was measured using a trypan blue assay. The IC_50_ values of kaempferol and its acetyl derivatives 3Ac-K and 4Ac-K for inhibition of cell growth were 46.7 μM, 42.2 μM, and 33.6 μM, respectively (Figure 5a). This indicated that 4Ac-K was 1.39-fold more effective in inhibiting cell proliferation than kaempferol. Conversely, 3Ac-K showed only a slight increase in potency compared to the parent compound. The IC_50_ values for quercetin and its acetyl derivatives 4Ac-Q and 5Ac-Q were 24.3 µM, 16.6 µM, and 17.4 µM, respectively (Figure 5b). Quercetin’s derivative 4Ac-Q exhibited a 1.46-fold improvement in cell growth inhibition compared to quercetin, whereas 5Ac-Q showed a 1.40-fold increase. Among the derivatives, 4Ac-Q consistently demonstrated higher cell growth inhibition within the range of 20–120 μM. Similarly, the IC_50_ values for myricetin and its acetyl compounds 5Ac-M and 6Ac-M were 27.2 µM, 47.3 µM, and 50.9 µM (Figure 5c). Acetylation decreased the inhibitory potency of myricetin compared to the parent compound, regardless of the presence or absence of the hydroxy group at the 5-position. The fold decreases were 1.74-fold for 5Ac-M and 1.87-fold for 6Ac-M. Figure 5d showed that the IC_50_ values for apigenin and its acetyl compounds 2Ac-A and 3Ac-A were 27.1 µM, 16.7 µM, and 31.1 µM. The 2Ac-A derivative exhibited a 1.62-fold enhancement in inhibitory effect compared to apigenin, while 3Ac-A showed a decrease in efficacy by 1.15-fold.

The IC_50_ values indicated that acetylation at the 5-position hydroxy group generally decreased the inhibitory potency of the flavonoids. For kaempferol, 4Ac-K (5-acetylated) was more effective than the parent compound, suggesting an exception. In contrast, quercetin (4Ac-Q vs. 5Ac-Q), myricetin (5Ac-M vs. 6Ac-M), and apigenin (2Ac-A vs. 3Ac-A) all showed that retaining the 5-position hydroxy group enhanced cell growth inhibition compared to their 5-acetylated derivatives. Thus, the presence of the 5-position hydroxy group generally enhances the inhibitory activity against cell growth.

### 2.7. Effects of 5-Position Hydroxyl Group Acetylation on Anti-Migration Activity

**Figure A1:**
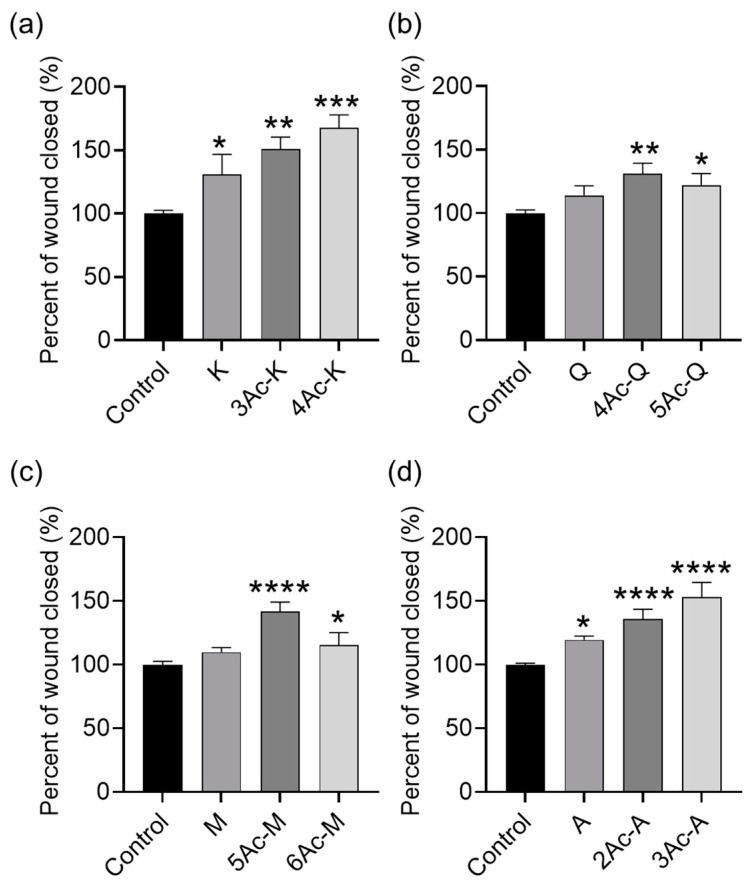
Assessment of anti-cellular migration using wound healing assay. Wound healing was quantified after 6 h of treatment with concentrations of (**a**) kaempferol, (**b**) quercetin, (**c**) myricetin, (**d**) apigenin and their acetyl derivatives at 2× IC_50_ values. The results shown (mean ± SD) are representative of two independent experiments (*n* = 9). * mark denoted significant differences: * *p* < 0.005, ** *p* < 0.01, *** *p* < 0.001, **** *p* < 0.0001 between control and each compound. Statistically analyzed by Dunnett’s multiple comparison test.

Next, the effects of acetylation of the hydroxyl group at 5-position on the antimigration activity of each flavonoid were evaluated using a wound healing assay. As depicted in Figure A1, kaempferol and apigenin exhibited statistically significant anti-migration activity in their parent forms, which was further enhanced upon acetylation. In contrast, quercetin and myricetin did not show significant anti-migration activity at double their IC_50_ concentrations, but both demonstrated significant enhancement in anti-migration effects upon acetylation. Notably, 4Ac-K and 3Ac-A, which have the 5-position acetylated, and 4Ac-Q and 5Ac-M, which retain the 5-position hydroxy group, exhibited the highest anti-migration effects within their respective groups. Thus, the results suggest that there is no clear correlation between the presence or absence of acetylation at the hydroxy group in position 5 and increased anti-migration activity.

**Figure 6:**
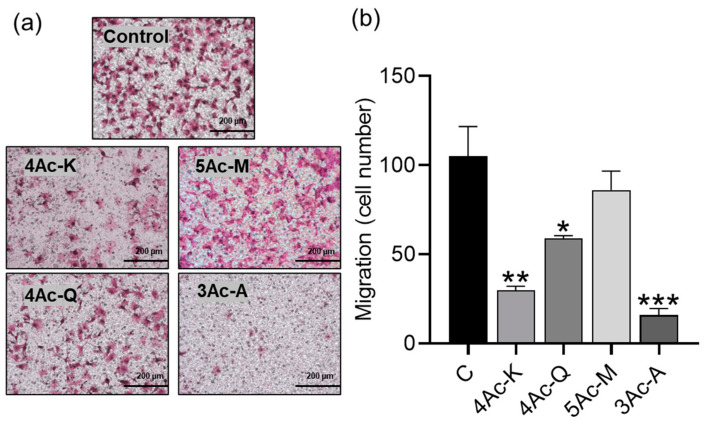
Assessment of anti-cellular migration using transwell assay. (**a**): Representative images from transwell chamber assay depicting migration by MDA-MB-231 cells treated for 24 h with DMSO (control) or the indicating samples. (**b**): Quantitation of migrated MDA-MB-231 cells from experiment shown in panel (**a**). Ability to inhibit cell migration was evaluated at half the IC_50_ concentration of each compound. The microscope magnification was 20× and the scale bar indicates 200 μm. Experiment was repeated at least three times in triplicate and combined results were shown as mean ± SD (*n* = 9). Significantly different (*p* < 0.05) compared with corresponding DMSO-treated control and each sample. * *p* < 0.05, ** *p* < 0.01, *** *p* < 0.001: significant differences between control and each compound by Dunnett’s multiple comparison test.

Each acetyl derivative that demonstrated excellent anti-invasive capacity in the wound healing assay was further evaluated in a transwell migration assay, measuring the effect of 24 h treatment with concentrations as low as half the IC_50_ concentration. This was done to determine whether these compounds could exhibit anti-migratory effects even at lower concentrations and shorter treatment durations (Figure 6). The results indicated that 3Ac-A exhibited the highest anti-migratory capacity, with a migration rate of 15.8% compared to the control. Conversely, 5Ac-M showed the lowest anti-migratory ability, with a migration rate of 86%. Comparing the acetyl derivatives of kaempferol, quercetin, and myricetin, which have flavonol as their basic backbone, 4Ac-K, 4Ac-Q, and 5Ac-M showed the highest anti-migration ability, in that order, respectively. This was consistent with the results of the wound healing assay.

### 2.8. Acetylated Flavonoids Induce Apoptosis

The apoptosis-inducing ability of 4Ac-K, 4Ac-Q, 5Ac-M, and 3Ac-A on MDA-MB-231 cells was evaluated by flow cytometry. The results of the anti-cell migration assay using a transwell assay, in which cells were treated with half the IC_50_ concentration for 24 h, showed statistically significant anti-migration effects for 3Ac-A, 4Ac-K, and 4Ac-Q, in that order. Consequently, cells were treated with half the IC_50_ value of each compound for 24 h to assess apoptosis induction, followed by Annexin/PI staining.

**Figure 7:**
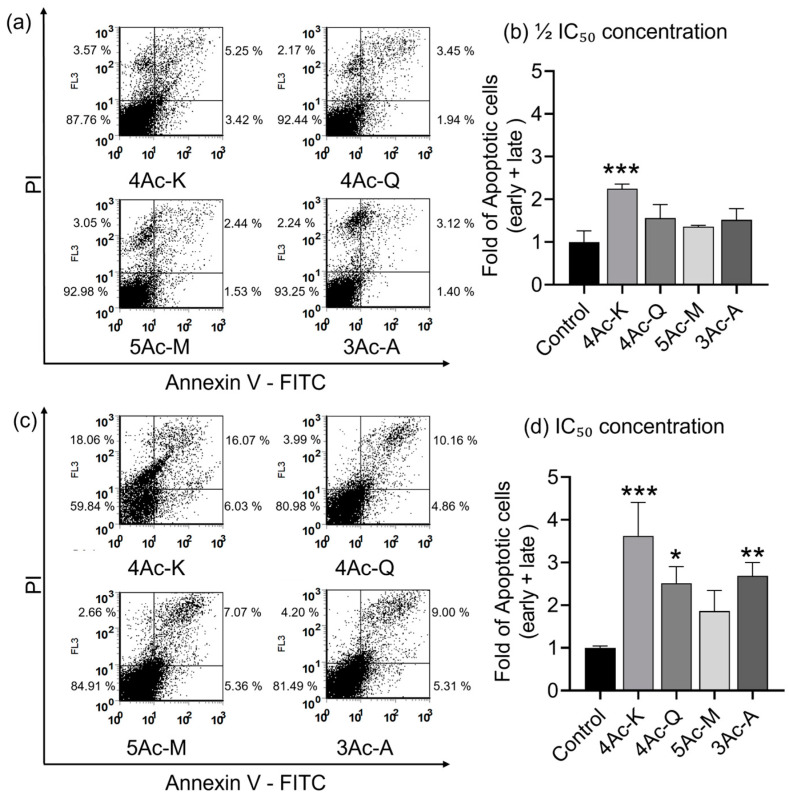
Effect of acetylated kaempferol, quercetin, myricetin, and apigenin on the apoptosis induction in MDA-MB-231 cells. (**a**,**c**) Representative flow histograms depicting the apoptotic fraction. Quantitation of % apoptotic fraction (early + late apoptotic cells) in MDA-MB-231 cells. Measurements in (**b**,**d**) are for a 24 h treatment at half the IC_50_ concentration and IC_50_, respectively. Data represented as the means ± SD; * mark denotes significant differences (* *p* < 0.005, ** *p* < 0.01, *** *p* < 0.001) between the control and each sample. Statistically analyzed by Dunnett’s multiple comparison test.

At half the IC_50_ dose, 4Ac-K exhibited the highest ability to induce apoptosis, at 2.2-fold that of the control (Figure 7b). In contrast, 3Ac-A, 4Ac-Q, and 5Ac-M showed no significant difference compared to the control. Since 3Ac-A and 4Ac-Q, which demonstrated significant effects in the anti-migration assay, did not induce significant apoptosis at half the IC_50_ dose, cells were also treated with the IC_50_ concentration for 24 h to determine if higher concentrations would induce apoptosis. At this concentrations, 4Ac-K maintained the strongest apoptosis-inducing ability, followed by 3Ac-A and 4Ac-Q, all of which showed significant apoptosis induction relative to the control (Figure 7d). Although 5Ac-M induced apoptosis, it did not significantly differ from the control.

While Figure 6b showed that 3Ac-A had stronger anti-migration ability against MDA-MB-231 cells compared to other acetyl derivatives, it did not show significant apoptosis induction at the same concentration (Figure 7b). This suggested that 3Ac-A possesses excellent antimigration activity independent of its apoptosis-inducing ability. Consequently, a comparison of anti-migration and apoptosis-inducing activities was conducted using 2Ac-A and 3Ac-A (Figure S1). The results indicated that 2Ac-A, treated at the IC_50_ concentration for 48 h, had superior apoptosis-inducing ability compared to 3Ac-A, while it exhibited inferior anti-migration ability. This strengthens the hypothesis that 3Ac-A, unlike other acetyl derivatives, has antimigration ability independent of apoptosis induction.

## 3. Discussion

### 3.1. Cell Proliferation Inhibitory Ability and Structure–Activity Relationships

As shown in Figure S1, all cell proliferation assays demonstrated that the flavonoids, excluding myricetin, inhibited proliferation in a dose-dependent manner. Their acetylated forms also exhibited similar dose-dependent inhibition patterns, albeit with varying degrees of potency. Of particular interest were the distinct growth inhibition patterns observed for myricetin and acetylated myricetin in the MDA-MB-231 cell line upon treatment with the acetylated flavonoids. Myricetin inhibited proliferation rapidly at 40 µM, and at 80 µM, the cells became round, aggregated, and floating, while acetylated myricetin showed a slow sigmoidal pattern of inhibition down to 80 µM. One of the major differences between myricetin and the other flavonoids used in this study is the presence of three hydroxyl groups on the B ring. As a result, acetylated myricetin has a bulkier steric structure compared to other flavonoids and undergoes a significant change in polarity. However, considering the consistent dose-dependent growth inhibition patterns observed in the HCT-116 and HepG2 cells, it is unlikely that the increased steric hindrance due to acetylation is the direct cause of the change in the growth inhibition pattern. The enhanced antitumor effects of quercetin-3-*O*-glucoside acylated with long-chain fatty acids suggest that the anticancer activity is influenced not only by the drug’s lipophilicity but also by its affinity for cellular receptors [56]. Therefore, it is plausible that the number of acetyl groups on the B ring of acetylated flavonoids may have led to chemical and physical interactions specific to the cell type. Cho et al. reported the differing metabolism of quercetin derivatives (with *O*-acylated pivaloxymethyl groups) in HCT-116 and MCF-7 breast cancer cells; it is important to consider the differences in the types and expression levels of metabolic enzymes and metabolic pathways in individual cells. To elucidate these hypotheses, it is necessary to (1) vary the number of acetyl modifications on the hydroxyl groups of the B ring of myricetin, (2) measure the cellular uptake of acetylated myricetin, and (3) track the metabolites of acetylated myricetin in each cell type.

Numerous reports suggest that the acetylation of flavonoids enhances cellular uptake, leading to an inhibitory effect on cancer cell growth. For instance, our study demonstrated an increased uptake of quercetin into HepG2 cells upon quercetin acetylation [41], and Lambert et al. observed an increased cellular uptake and an enhanced growth inhibition in HCT-116 cells upon EGCG acetylation [57]. However, our present study revealed exceptions to this trend. In certain cell types and with specific flavonoids, the cell proliferation inhibitory effect decreased after acetylation, depending on the cell type. These findings are summarized in Table 2.

The strength of cell proliferation inhibitory effects was ranked as follows: apigenin > kaempferol > naringenin. Similarly, luteolin showed stronger cell proliferation inhibitory effects compared to quercetin and taxifolin. Importantly, the order of inhibitory activity did not change after acetylation for any of these flavonoids.

Interestingly, both apigenin and luteolin derivatives containing chrysin exhibited decreased inhibitory activity upon acetylation in the MDA-MB-231 and HCT-116 cells. Conversely, acetylation of flavonols, except for myricetin in the MDA-MB-231 cells, contributed to enhanced inhibitory activity. All flavonols showed enhanced activity upon acetylation, particularly the cell proliferation inhibitory activity of taxifolin, which was reduced to a minimum of 125.8 μM by acetylation, even though the half-life inhibitory concentration (IC_50_) was above 160 μM in all cell types. Furthermore, comparison of the IC_50_ values in Figure 5 suggested that for many flavonoids, avoiding acetylation of the 5-OH group in the A ring may help maintain their activity. The structural difference between flavonols and flavones lies in the presence or absence of a hydroxyl group at the C-3 position of the C ring. It has been reported that the absence of a hydroxyl group at this position enhances cell proliferation inhibitory effects [58,59]. This supports our findings that luteolin, compared to quercetin, and apigenin, compared to kaempferol, inhibit the proliferation of various cancer cell types at lower IC_50_ values. Furthermore, acetylation of the hydroxyl group at C-3 in the C ring resulted in enhanced cell proliferation inhibitory activity. Consequently, the compounds acetylated at the C-3 hydroxyl group, such as 4Ac-K and 5Ac-Q, showed no significant difference in IC_50_ values compared to the compounds lacking the C-3 hydroxyl group, such as 3Ac-A and 4Ac-L. This suggests that acetylating the hydroxyl group at C-3 in the C ring to a non-hydroxyl group enhances growth inhibitory activity. Consistent with this observation, studies by Zhang et al. [60] and Karancsi et al. [61] have demonstrated that methylation at the C3 position of the C ring plays a crucial role in enhancing the growth inhibitory activity of Rhamnetin (3′,7-dimethylquercetin) and 3-*O*-methylquercetin. On the other hand, apigenin and naringenin, as well as quercetin and taxifolin, share differences in the presence or absence of a C2–C3 double bond. The presence of the C2–C3 double bond is crucial for the inhibitory activity of flavonoids. Studies have demonstrated that quercetin and taxifolin, when evaluated in human leukemia HL-60 cells, exhibit higher proliferation inhibitory activity when the C ring contains a double bond C-ring [62]. Additionally, it has been reported that the presence of a C2–C3 double bond in citrus-derived flavonoids is essential for exerting anticancer effects against cancer cells [63]. Our findings are consistent with these observations. Taxifolin, a flavanone, lacks a C2–C3 double bond, making it susceptible to inactivation through strong hydrogen bonding with polymers [64]. Our study suggests that this inactivation may be mitigated by acetylation. It is conceivable that protection of hydroxyl groups by acetylation disrupts interactions via hydrogen bonding. However, considering the possibility of deacetylation within cells followed by inactivation, the cell proliferation inhibitory effects might result from interactions as acetylated derivatives with receptors on cell membranes. Future studies should evaluate interactions between acetylated flavonoids and receptors before deacetylation induction.

From these results, it is suggested that to enhance the cell proliferation inhibitory activity through acetylation, attention should be paid to the acetylation of the C-3 hydroxyl group and the absence of acetyl groups at the 5-OH position in the A ring. However, as shown in Table 2, there are numerous exceptions to these observations, including cell specificity. To comprehensively understand the delicate effects of acetylation on flavonoid activity, further studies are needed to (1) expand the types of flavonoids targeted for acetylation, (2) increase the diversity of cell types evaluated, (3) measure the impact on cellular uptake and metabolism, and (4) elucidate the interactions of acetylated derivatives with cell membrane receptors. Such research will strengthen our understanding of the nuanced effects of acetylation on flavonoid activity. 

### 3.2. Effects of Acetylation of Quercetin and Kaempferol on the Cell Cycle

Flavonoids are known to possess cell proliferation inhibitory properties, and this mechanism has been reported in multiple studies [65,66,67].

In MDA-MB-231 cells, kaempferol induced an increase in CyclinB1 and p-Cdc2 levels, along with a decrease in CyclinD1. This suggests that kaempferol caused a G2/M phase arrest via inhibition of Cdc2, leading to the reduced CyclinD1 levels associated with the G1 phase. Consistent with this, a study by Zhu et al. reported a decrease in G1 phase cells and an increase in G2/M phase cells over time upon the addition of kaempferol to MDA-MB-231 cells [68]. Comparison of 4Ac-K with the controls revealed a decrease in CyclinD1 levels and an increase in CyclinA and CyclinB1 at 12 h, suggesting a potential influence on cell cycle progression. Previous studies have indicated that the inhibition of the nuclear translocation of the CyclinB1/Cdc2 complex prolongs G2 phase duration [69]. A regulator of these processes is 14-3-3σ; thus, detecting 14-3-3σ would be essential to ascertain whether the observed increase in CyclinB1 following 4Ac-K treatment correlates with cell cycle delay.

Quercetin treatment for 12 h led to decreased CyclinD1 and CyclinE levels, while CyclinB and p-Cdc2 levels showed no significant change. Chekuri et al. treated MDA-MB-231 cells with 10 µg/mL of quercetin extracted from A. indica plant leaves for 24 h, reporting an increase in the G1 phase [70]. Our findings diverge, suggesting quercetin’s 24 h treatment affects the G2/M phase in MDA-MB-231 cells. The disparity might stem from using pure quercetin in our study versus plant extracts in Chekuri et al., potentially explaining different effects on the cell cycle. Leong et al. reported no significant cell cycle impact with 24 h treatment of 200 µM quercetin compared to controls [71]. This disparity in results could be attributed to their use of a higher concentration of quercetin than ours and their utilization of flow cytometry for cell cycle quantification.

The 12 h treatment of 5Ac-Q mirrored the findings seen with 4Ac-K, implying a potential G2/M phase arrest mediated by CyclinB1 and CyclinA accumulation through p-Cdc2 inactivation. Acetylation altered the dynamics of proteins such as CyclinD1 and CyclinB1, suggesting their involvement in cell cycle arrest and regulation, independent or dependent of their behavior in parent compounds.

In HCT-116 cells, the addition of kaempferol for 12 h resulted in an increased expression of CyclinB1, which is involved in the progression from G2 to M phase, while p-Cdc2 expression levels were comparable to those of controls. The increased expression of CyclinD1 also suggests that progression to the G1 phase is taking place, as Budisan et al. reported similar results, in which the addition of kaempferol to HCT-116 cells for 24 h reduced the percentage of cells in the G1 phase and increased the number of cells in the G2 phase [72]. This was consistent with our results for 24 h treatment with kaempferol. The addition of 4Ac-K to HCT-116 cells resulted in decreased CyclinD1 and CyclinB1 levels and increased phosphorylated inactive Cdc2, unlike the parent compound, indicating a potential arrest of the cell cycle in the G2/M phase. Chiou et al. reported that treatment with acetylated EGCG (AcEGCG) activates ERK, induces Cdc 25A degradation, and inhibits cyclin B1/CDK1 complex formation [73]. Similarly, at 12 h and 24 h, quercetin and 5Ac-Q increased CyclinB1 and the inactive form, p-Cdc2, suggesting that Cdc2 inactivation is accompanied by increased accumulation of CyclinB1 in the cytosol. These results indicate that all samples in HCT-116 affect the G2/M phase, with the acetyl compound possibly having a stronger effect on the G2/M phase than the parent compound.

For HepG2 cells, 4Ac-K exhibited increased phosphorylated Cdc2 (p-Cdc2) levels, along with decreased CyclinD1 levels after 12 h compared to kaempferol, indicating a pronounced effect on the G2/M phase.

Quercetin and 5Ac-Q treatments for 12 and 24 h resulted in decreased CyclinD1 and CyclinB1 levels, as well as increased p-Cdc2 levels, which also led to a slight decrease in CyclinA levels associated with the S phase. This suggests a potential impact on the G2/M phase, consistent with findings from Xu et al., who reported that 24 h treatment with 10 µM quercetin increased the proportion of cells in the G2/M phase [74]. Similar studies have shown that acylated quercetin-3-*O*-glucoside increased the number of cells in the G2/M phase in HepG2 cells [56]. Additionally, our results indicate that 5Ac-Q may affect the G2/M phase more strongly than quercetin. Furthermore, our previous study demonstrated a delayed metabolite effect of quercetin and 4Ac-Q in HepG2 cells after 6 h, indicating an enhanced uptake [41]. Together, these findings suggest distinct effects of quercetin and 5Ac-Q treatments on the cell cycle.

Treatment of MDA-MB-231 cells with quercetin and 5Ac-Q and of HCT-116 cells with kaempferol and 4Ac-K for 12 h suggested varying effects on the cell cycle. Treatment of HCT-116 and HepG2 cells with 5Ac-Q indicated a stronger impact on the G2/M phase compared to treatment with quercetin. These findings underscore one outcome supporting the enhanced cell proliferation inhibition by acetylation of quercetin and kaempferol. While the influence of acetylation on the cell cycle of each flavonol was suggested, further studies are needed to accurately correlate the differences in cell proliferation inhibition between parent compounds and acetylated derivatives with cell cycle arrest. The quantification of cell numbers in each phase using flow cytometry, along with the comparison of the metabolites of the acetylated compounds in cells compared to those of their parent compounds, will be essential to achieve a more definitive understanding.

### 3.3. Anti-Migration and Apoptosis-Inducing Activities of Acetylated Flavonoids

In the assessment of anti-migration activity using a wound healing assay and a migration assay, the acetyl derivatives containing apigenin exhibited notable anti-migration effects. Apigenin has shown the inhibition of IL-6 in MDA-MB-231 cells and the suppression of metastasis by hindering HGF-promoted invasive growth [75,76,77]. Similarly, in this study, an acetyl derivative of kaempferol and quercetin also displayed superior migration inhibitory activity compared to its parent compound. Quercetin and kaempferol, parent compounds, have been reported to impede breast cancer cell invasion notably by inhibiting MMP-3 [78]. As there are currently no reports on the mechanisms of action for the anti-migration effects of acetylated flavonoids, it would be most effective to base future comparative studies on the known mechanisms of their parent compounds.

Notably, 4Ac-K exhibited potent apoptosis induction at low doses, suggesting a possible correlation between its anti-migration activity and apoptosis induction. In this study, both 2Ac-A and 4Ac-K showed markedly superior apoptosis induction and anti-migration ability. The mechanism by which apigenin induces apoptosis may also be related to anti-angiogenesis and anti-migration in human hepatocellular carcinoma cells [79]. However, the findings from Figure 6, Figure 7 and Figure S1 suggest that 3Ac-A may have anti-migration properties independent of its apoptosis-inducing ability. Further studies with apoptosis inhibitors and related agents are needed to clarify whether 3Ac-A operates through pathways independent of apoptosis induction. The reported anti-metastatic effects of natural compounds such as isothiocyanates, achieved by downregulation of the IAP family, are noteworthy [80]. Our previous studies have shown that 4Ac-Q strongly downregulates the IAP family [23,41], suggesting a possible link between IAP family regulation and anti-migration effects. Given this possibility, it is plausible that acetylated apigenin derivatives may also regulate the IAP family. Investigating this potential relationship could help clarify the connection between IAP family regulation and the anti-migration effects of these compounds. However, it should also be noted that apigenin induces apoptosis through various pathways other than caspase-3 activation [58]. By elucidating these additional mechanisms, a more comprehensive understanding of the apoptosis-inducing and anti-migratory pathways of apigenin, 2Ac-A, and 3Ac-A may be achieved.

### 3.4. Anti-Migration Activity and Structure–Activity Relationships

The compounds with enhanced anti-migratory activity upon acetylation included 4Ac-K, 5Ac-Q, 6Ac-M, and 5Ac-T. Similar to the results of the inhibitory effect on cell proliferation, the acetylation modification at the C-ring 3 position may also contribute to the enhancement of anti-migratory activity. Yamauchi et al. reported that methylation of the hydroxyl group at position 3 of the C ring of quercetin inhibited the migration ability of B16 melanoma cells [25]. One of the primary contributions of the acetyl group is likely the protection of the hydroxyl group at position 3. Additionally, the fact that acetylated derivatives of apigenin, which lacks a hydroxyl group at position 3, exhibited significantly increased activity suggests that there may be further contributions beyond the protection of the 3-position hydroxyl group. Elucidating these additional contributions will be one of the most important research tasks in the future, as 3Ac-Q demonstrated anti-migratory effects even at concentrations that did not induce apoptosis.

Comparing 4Ac-K with 5Ac-Q and 6Ac-M, 4Ac-K showed the highest anti-migration effect. Similarly, among 2Ac-C, 3Ac-A, and 4Ac-L, 3Ac-A demonstrated the highest migration inhibition. Thus, the presence of an acetyl group at the 4′-position of the B ring contributed to the enhanced anti-migration activity. However, the slightly reduced inhibitory effect of 3Ac-N, whose parent compound is naringenin with a 4′-OH group, suggests that a C2–C3 double bond is required as a basic backbone. This experiment alone could not determine the effect of the presence or absence of a hydroxyl group at position 5 of the A ring on anti-migration ability. It is suggested that further insights may be obtained by synthesizing and comparing new compounds, such as acetylated derivatives (3Ac-L and 2Ac-N), in which the hydroxyl group at position 5 of luteolin and naringenin remains.

In terms of the contribution of acetylation to the enhanced induction of apoptosis, the comparison among 4Ac-K, 4Ac-Q, and 5Ac-M suggests that acetyl modification at position 4′ of the B ring is likely responsible for the increased activity. Specifically, compounds like 2Ac-A and 4Ac-K, which exhibited significant apoptosis induction, share the structural feature of having an acetyl group only at position 4′ of the B ring, presumed to enhance apoptosis induction, at least in the context of MDA-MB-231 cells. Furthermore, comparing the apoptosis-inducing abilities of 2Ac-A and 3Ac-A (Figure S2) revealed that the presence of a hydroxyl group at position 5 of the A ring is necessary, consistent with the results observed in the evaluation of cell proliferation inhibition.

## 4. Materials and Methods

### 4.1. Chemicals, Antibodies, and Reagents

Quercetin for the synthesis of 4Ac-Q (purity ≥ 90%) was purchased from Sigma-Aldrich (St. Louis, MO, USA), and quercetin for the bioactivity assay (purity > 99%) was purchased from LKT Laboratories (St. Paul, MN, USA). Kaempferol, Myricetin, Chrysin, and Naringenin were purchased from TCI Co., Ltd. (Tokyo, Japan). Apigenin and luteolin were obtained from Wako Co., Ltd. (Osaka, Japan) and Funakoshi (Tokyo, Japan), respectively. Trypan blue was purchased from Nacalai Tesque (Tokyo, Japan). Hematoxylin-Eosin Stain kit was from Cosmo Bio Co., Ltd. (Tokyo, Japan). The antibodies against Cyclin A1 (sc-271682), Cyclin B1 (sc-245), Cyclin D1 (sc-20044), Cyclin E (sc-377100), P21 (sc-756), P27 (sc-528), and p-Cdc2 p34 (sc-136014) were obtained from Santa Cruz Biotechnology (Dallas, TX, USA). Chk1 (#2360), p-chk1 (#2348), and anti-rabbit (#7074) secondary antibodies were from Cell Signaling Technology (Beverly, MA, USA). The antibody against β-actin (A5441) was from Sigma-Aldrich (St. Louis, MO, USA). Transwell Permeable Support (8 mm polycarbonate membrane) chambers used for cell migration assay were purchased from Corning (Corning, NY, USA).

### 4.2. Synthesis of Acetylated Derivatives of Flavonoids

#### 4.2.1. 3,5,7,4′-. O-tetraacetate kaempferol (4Ac-K) (Compound **2**)

Kaempferol (**1**) (0.480 g, 1.67 mmol) was dissolved in pyridine (4 mL), acetic anhydride (5 eq, 0.8 mL) was added, and the solution was stirred at room temperature for 12 h. After the reaction was completed, 30 mL of cold water was added to the stirred solution to quench the reaction. The resulting precipitate was collected by filtration and dried overnight. For purification, each filtered precipitate was dissolved in 50 mL of methanol with heating. The solution was then filtered, cooled, and recrystallized. After recrystallization, HPLC was performed to confirm the purity of the obtained compounds. Compounds with a purity of more than 97% were used.

#### 4.2.2. 3,5,7,3′,4-. O-pentaacetate quercetin (5Ac-Q) (Compound **4**)

Quercetin (**3**) (0.638 g, 2.11 mmol) was dissolved in pyridine (4 mL), acetic anhydride (6 eq, 1.2 mL) was added, and the solution was stirred at room temperature for 1 h. After the reaction was completed, cold water (30 mL) was added to the stirred solution to quench the reaction. The resulting precipitate was collected by filtration and dried overnight. For purification, each filtered precipitate was dissolved in methanol (75 mL) with heating. The solution was then filtered, cooled, and recrystallized. After recrystallization, HPLC was performed to confirm the purity of the obtained compounds. Compounds with a purity of more than 97% were used.

#### 4.2.3. 3,5,7,3′,4′,5′-. O-hexaacetate myricetin (6Ac-M) (Compound **6**)

Myricetin (**5**) (0.498 g, 1.56 mmol) was dissolved in pyridine (4 mL), acetic anhydride (7 eq, 1.1 mL) was added, and the solution was stirred at room temperature for 12 h. After the reaction was completed, cold water (30 mL) was added to the stirred solution to quench the reaction. The resulting precipitate was collected by filtration and dried overnight. For purification, each filtered precipitate was dissolved in methanol (150 mL) with heating. The solution was then filtered, cooled, and recrystallized. The recrystallized product was collected and used as a sample.

#### 4.2.4. 5,7-. O-diacetate chrysin (2Ac-C) (Compound **8**)

Chrysin (**7**) (0.502 g, 1.97 mmol) was dissolved in pyridine (8 mL), acetic anhydride (3 eq, 0.555 mL) was added, and the solution was refluxed for 7 h. After the reaction was completed, cold water (50 mL) was added to the stirred solution to quench the reaction. The resulting precipitate was collected by filtration and dried overnight. For purification, each filtered precipitate was dissolved in methanol (100 mL) with heating. The solution was then filtered, cooled, and recrystallized. The recrystallized product was collected and used as a sample.

#### 4.2.5. 5,7,4′-. O-triacetate apigenin (3Ac-A) (Compound **10**)

Apigenin (**9**) (0.4868 g, 1.80 mmol) was dissolved in pyridine (12 mL), acetic anhydride (4 eq, 0.680 mL) was added, and the solution was refluxed for 7 h. After the reaction was completed, cold water (50 mL) was added to the stirred solution to quench the reaction. The resulting precipitate was collected by filtration and dried overnight. For purification, each filtered precipitate was dissolved in methanol (150 mL) with heating. The solution was then filtered, cooled, and recrystallized. The recrystallized product was collected and used as a sample.

#### 4.2.6. 5,7,3’,4′-. O-tetraacetate luteolin (4Ac-L) (Compound **12**)

Luteolin (**11**) (0.496 g, 1.73 mmol) was dissolved in pyridine (20 mL), acetic anhydride (5 eq, 0.810 mL) was added, and the solution was refluxed for 7 h. After the reaction was completed, cold water (150 mL) was added to the stirred solution to quench the reaction. The resulting precipitate was collected by filtration and dried overnight. For purification, each filtered precipitate was dissolved in methanol (150 mL) with heating. The solution was then filtered, cooled, and recrystallized. The recrystallized product was collected and used as a sample.

#### 4.2.7. 5,7,4′-. O-triacetate naringenin (3Ac-N) (Compound **14**)

Naringenin (**13**) (0.511 g, 1.87 mmol) was dissolved in pyridine (27 mL), acetic anhydride (100 eq, 17.5 mL) was added, and the solution was stirred at room temperature for 7 h. After the reaction was completed, cold water (150 mL) was added to the stirred solution to quench the reaction. The resulting precipitate was collected by filtration and dried overnight. For purification, each filtered precipitate was dissolved in methanol (50 mL) with heating. The solution was then filtered, cooled, and recrystallized. The recrystallized product was collected and used as a sample.

#### 4.2.8. 3,5,7,3,’4′-. O-pentaacetate taxifolin (5Ac-T) (Compound **16**)

Taxifolin (**15**) (0.187 g, 0.614 mmol) was dissolved in pyridine (27 mL), acetic anhydride (8 eq, 0.46 mL) was added, and the solution was stirred at 37 °C for 1 h. After the reaction was completed, cold water (20 mL) was added to the stirred solution to quench the reaction. The resulting precipitate was collected by filtration and dried overnight. For purification, each filtered precipitate was dissolved in methanol (30 mL) with heating. The solution was then filtered, cooled, and recrystallized. The recrystallized product was collected and used as a sample.

#### 4.2.9. 3,7,4′-. O-triacetate kaempferol (3Ac-K) (Compound **17**)

Kaempferol (**1**) (0.106 g, 1.67 mmol) was dissolved in pyridine (4 mL), acetic anhydride (3 eq, 0.104 mL) was added, and the solution was stirred at room temperature for 7 min. After the reaction was completed, cold water (30 mL) was added to the stirred solution to quench the reaction. The resulting precipitate was collected by filtration and dried overnight. For purification, each filtered precipitate was dissolved in methanol (30 mL) with heating. The solution was then filtered, cooled, and recrystallized. The recrystallized product was collected and used as a sample.

#### 4.2.10. 3,7,3′,4′-. O-tetraacetate quercetin (4A-Q) (Compound **18**)

Quercetin (**3**) (0.600 g, 1.98 mmol) was dissolved in pyridine (4 mL), acetic anhydride (4.2 eq, 0.79 mL) was added, and the solution was stirred at room temperature for 12 min. After the reaction was completed, cold water (30 mL) was added to the stirred solution to quench the reaction. The resulting precipitate was collected by filtration and dried overnight. For purification, each filtered precipitate was dissolved in methanol (50 mL) with heating. The solution was then filtered, cooled, and recrystallized. The recrystallized product was collected and used as a sample.

#### 4.2.11. 3,7,3′,4′,5′-. O-pentaacetate myricetin (5Ac-M) (Compound **19**)

Myricetin (**5**) (0.346 g, 1.09 mmol) was dissolved in pyridine (4 mL), acetic anhydride (5 eq, 0.51 mL) was added, and the solution was stirred at room temperature for 7 min. After the reaction was completed, cold water (30 mL) was added to the stirred solution to quench the reaction. The resulting precipitate was collected by filtration and dried overnight. For purification, each filtered precipitate was dissolved in methanol (150 mL) with heating. The solution was then filtered, cooled, and recrystallized. The recrystallized product was collected and used as a sample.

#### 4.2.12. 7,4′-. O-diacetate apigenin (2Ac-A) (Compound **20**)

Apigenin (**9**) (0.0314 g, 0.116 mmol) was dissolved in pyridine (4 mL), acetic anhydride (2 eq, 0.022 mL) was added, and the solution was stirred on ice for 5 min. After the reaction was completed, cold water (50 mL) was added to the stirred solution to quench the reaction. The resulting precipitate was collected by filtration and dried overnight. For the purification of acetylated apigenin, it was dissolved in acetone. The solution was then concentrated using an evaporator. Hexane was added, and the mixture was left overnight to crystallize.

### 4.3. Structure Identification of Acetylated Flavonoids

All acetylated flavonoids were determined by ^1^H-NMR recorded on a spectrometer (JEOL ECA-600, 600 MHz for 1H, JEOL, Tokyo, Japan) using tetramethylsilane (TMS) as an internal standard. All acetylated flavonoids except 2Ac-A were determined using Chloroform-D1, and 2Ac-A was measured using Acetone-D6 as the solvent. The infrared absorption wavelengths of each compound were measured using an FT-IR/IRT-3000 ATR-30-Z spectrophotometer (Japan Spectroscopic Corporation, Tokyo, Japan). Attenuated total reflection (ATR) was performed by placing a powder sample on the entire surface of the ATR crystal and pressing the sample hard against the prism while compressing it. The measuring range was 400–4000 cm^−1^. The purity of each compound was confirmed by HPLC to be at least 97%.

### 4.4. Cell Culture

The human breast cancer cell line MDA-MB-231 was obtained from the American Type Culture Collection (Manassas, VA, USA) and growth conditions were optimized at 37 °C in a 5% CO_2_ atmosphere. Cells were cultured in Roswell Park Memorial Institute medium (RPMI 1640) supplemented with 10% fetal bovine serum, 1% penicillin-streptomycin-neomycin (PSN), and 1.5% glutamine. The human hepatocellular carcinoma cell line HepG2 was obtained from the RIKEN Cell Bank (RCB1648) ( Tsukuba, Ibaraki, Japan), and the human colon carcinoma cell line HCT-116 was obtained from the American Type Culture Collection. These cells were cultured in DMEM medium supplemented with 10% fetal bovine serum and 1% penicillin-streptomycin-glutamine (PSG) at 37 °C in a 5% CO_2_ atmosphere. All cells were treated with the indicated time and dose samples after 24 h of culture.

### 4.5. Cell Viability Assay

Cell viability was determined by trypan blue dye exclusion assay: MDA-MB-231 (5.0 × 10^4^ cells/well), HCT-116 (1.96 × 10^5^ cells/well) or HepG2 (2.4 × 10^5^/well) cells were plated in each well of 6-well plates. Cells were treated with each flavonoid and acetylated flavonoids for 48 h. After cell collection by trypsin, each cell solution was stained with 100 μL of 0.4% trypan blue solution for 1 min. Cells were counted on a hemocytometer for viable cells, and cell viability was expressed as the optical density ratio of treatment to control.

### 4.6. Western Blot Analysis

The Western blot analysis procedure followed our previously established protocols [81]. Briefly, MDA-MB-231 (8.1 × 10^4^ cells/well), HCT-116 (2.27 × 10^5^ cells/well) or HepG2 (2.78 × 10^5^/well) cells were each plated in 6-well plates. Then, 1 mM hydroxyurea was added 24 h prior to sample addition and all cell cycles were synchronized in S phase. After 24 h, kaempferol, 4Ac-K, quercetin, and 5Ac-Q were treated with 40 μM. After 12, 24, and 48 h, cells were collected and lysed using RIPA buffer. RIPA buffer consisted of 50 mM Tris-HCl (pH 8.0), 150 mM NaCl, 1 mM EDTA, 1% Nonidet P-40, 0.25% Na-deoxycholate, 1 mM sodium fluoride, 1 mM sodium orthovanadate, 1 mM phenylmethylsulfhonyl fluoride, and a proteinase inhibitor cocktail (Nacalai Tesque, Kyoto, Japan). Equal concentrations of lysed proteins were subjected to SDS-PAGE and transferred electrophoretically to PVDF membranes (GE Healthcare UK, Amersham, Buckinghamshire, UK) for 2 h. After blocking, the membranes were incubated overnight at 4 °C with specific primary antibodies, then exposed to the corresponding HRP-conjugated secondary antibodies for 1 h at room temperature. Each membrane was then detected using chemiluminescence and was scanned and evaluated using LumiVision software (version 140.ex) (TAITEC, Saitama, Japan).

### 4.7. Detection of Apoptosis Using Flow Cytometry

Quantitation of apoptotic cell death was assessed by eBioscience^TM^ AnnexinV-FITC Apop Kit, purchased from Invitrogen-Life Technologies (Carlsbad, CA, USA). In brief, after 24 or 48 h of treatment with each sample, MDA-MB-231 cells were collected and suspended in binding buffer and FITC-Annexin V/PI stain; after 15 min of staining, cells were analyzed using flow cytometry at FL1 (530 nm) and FL3 (630 nm) (CyFlow^@^, Sysmex Partec GmbH, Gorlitz, Germany).

### 4.8. Wound Healing Assay

MDA-MB-231 cells were plated into each well of 6-well plate and the cells were cultured approximately confluent monolayer. Each well was scratched by 200 μL pipette tip and treated with desired concentration of samples. Cells were allowed to migrate for 6 h, then, the cells were removed from the medium, fixed by methanol, and stained by Giemsa stain solution (Sigma-Aldrich, St. Louis, MO, USA). Migration of cells was quantified by measuring distances between the borders of cells; image analysis was performed using Image J software (version 1.50i) (Bethesda, MD, USA). At least three nonoverlapping areas per well were examined for wound healing.

### 4.9. Cell Migration Assay

The migration ability of MDA-MB-231 was performed by using polycarbonate membrane transwell inserts (Corning, NY, USA). Membrane pore size was 8.0 μm. MDA-MB-231 (3.0 × 10^5^/well) cells were suspended in serum-free medium containing DMSO or the test agents and placed in the upper compartment of the 6-well type transwell chamber. After incubation for 24 h, the cells on the upper surface of the transwell were removed by cotton swab. The motile cells from the bottom face of the filter were fixed with methanol and stained with hematoxylin and eosin. The evaluation of migration was analyzed by taking a picture with KEYENCE microscope (BZ-X810, KEYENCE, Osaka Oosaka, Japan) at 20 objective magnifications and counting migrated cells.

### 4.10. Statistical Analysis

Statistical analysis of the data was determined by a one-way analysis of variance (ANOVA) test followed by Tukey’s multiple comparison test and Dunnett’s test as described in each figure legend, employing GraphPad Prism 9.5.1 software (San Diego, CA, USA). The data are expressed as the means ± standard deviation (SD). All experiments were performed in biological triplicates (*n* = 3) with at least three replicates. The significance level was set as *p*-values of less than 0.05.

## 5. Conclusions

Among the 12 acetylated flavonoids tested, the acetyl derivatives of kaempferol and quercetin showed enhanced cell proliferation inhibition in all three cancer cell types. The evaluation of anti-migratory and apoptosis-inducing activities against MDA-MB-231 cells suggested that apigenin may acquire a unique anti-migratory activity different from that of other flavonoids through acetylation.

The presence of a hydroxyl group at the 5 position of flavonoid structures has been shown to potentially enhance cell proliferation inhibition and antimigration activity through acetylation. However, further verification of this effect is needed across compounds. Acetylated flavonoids may need to be manufactured with the appropriate number and position of acetyl groups to maintain or enhance their biological activity. Furthermore, future research should focus on synthesizing a wider variety of acetylated flavonoids and accumulating more data on their cellular uptake patterns, metabolic rates, and metabolites.

## Figures and Tables

**Table 1:** Antiproliferative activity of flavonoids and acetyl flavonoids against cancer cells.

		Cell Line	
Compound	MDA-MB-231	HCT-116	HepG2
Flavonols (IC_50_ μM)			
K	46.7 ± 6.8	34.9 ± 1.0	33.4 ± 2.1
4Ac-K	33.6 ± 0.6	28.5 ± 0.5	23.2 ± 0.1
Q	24.3 ± 9.6	23.5 ± 2.8	28.2 ± 5.0
5Ac-Q	17.4 ± 10.8	15.7 ± 2.6	15.5 ± 0.2
M	27.2 ± 0.3	Over 160	Over 160
6Ac-M	50.9 ± 5.5	81.7 ± 36	76.6 ± 20.4
Flavone (IC_50_ μM)			
C	37.5 ± 5.0	27.5 ± 2.9	25.3 ± 2.8
2Ac-C	38.4 ± 5.4	32.2 ± 1.3	23.8 ± 9.0
A	27.1 ± 5.2	19.0 ± 4.2	25.8 ± 1.9
3Ac-A	31.1 ± 7.2	21.9 ± 2.6	6.5 ± 1.0
L	12.9 ± 3.6	9.3 ± 2.2	10.2 ± 2.0
4Ac-L	20.2 ± 6.3	12.2 ± 3.7	12.4 ± 3.5
Flavanone (IC_50_ μM)			
N	Over 160	120.4 ± 39.8	118.5 ± 30.8
3Ac-N	156.4 ± 23.1	94.3 ± 23.1	97.9 ± 10.6
T	Over 160	Over 160	Over 160
5Ac-T	128.0 ± 23.1	125.8 ± 23.1	152.2 ± 23.1

IC_50_ ± SD values in μM after 48 h incubation. IC_50_ values were calculated from dose-response curves.

**Table 2:** Increase/decrease in cell proliferation inhibition ability with acetylation.

Sample	MDA-MB-231	HCT-116	HepG2
Flavonols			
3Ac-K	↑	-	-
4Ac-K	↑	↑	↑
4Ac-Q	↑	-	-
5Ac-Q	↑	↑	↑
5Ac-M	↓	-	-
6Ac-M	↓	↑	↑
Flavone			
2Ac-C	↓	↓	↑
2Ac-A	↑	-	-
3Ac-A	↓	↓	↑
4Ac-L	↓	↓	↓
Flavanone			
3Ac-N	↑	→	→
5Ac-T	↑	↑	↑

Comparison results of IC_50_ values are indicated by the direction of the arrows; gray upward arrows indicate a slight increase. Horizontal arrows indicated no statistically significant change.

## Data Availability

The data used to support the findings of this study are available from the corresponding author upon request.

## Supplementary Materials

The following supporting information can be downloaded at: https://www.mdpi.com/article/10.3390/ijms25147689/s1.
