# Supplementary material for: Comparative Analysis of Acetylated Flavonoids’ Chemopreventive Effects in Different Cancer Cell Lines
Source: Int J Mol Sci. 2024 Jul 13;25(14):7689. doi: 10.3390/ijms25147689 (PMC11276853; doi:10.3390/ijms25147689)
Supplement: Supplementary file 1 [file ijms-25-07689-s001.zip › ijms-3067388-supplementary.pdf]

Table S1

^{1}H-NMR shift for Flavonoids of hydroxy group

	Kaempferol	Quercetin	Myricetin
C5-OH	12.48 (1H, s)	12.50 (1H, s)	12.50 (1H, s)
C7-OH	10.79 (1H, s)	10.79 (1H, s)	10.79 (1H, s)
C4′-OH	10.12 (1H, s)	9.61 (1H, s)	9.36 (1H, s)
C3-OH	9.41 (1H, s)	9.39 (1H, s)	8.83 (1H, s)
C3′/ 5′-OH	-	9.32 (1H, s)	9.23 (2H, d)
C5-OH	12.48 (1H, s)	12.50 (1H, s)	12.50 (1H, s)
	**Chrysin**	**Apigenin**	**Luteolin**
C5-OH	Not detectable	Not detectable	Not detectable
C7-OH	10.95 (1H, s)	10.86 (1H, s)	10.86 (1H, s)
C4′-OH	-	10.38 (1H, s)	9.96 (1H, s)
C3′-OH	-	-	9.44 (1H, s)
	**Naringenin**	**Taxifolin**	
C5-OH	12.15 (1H, s)	11.91 (1H, s)	
C7-OH	10.80 (1H, s)	10.84 (1H, s)	
C4′-OH	9.60 (1H, s)	9.00 (1H, s)	
C3-OH	-	9.06 (1H, s)	
C3′-OH	-	5.78 (1H, dd)	

(a) MDA-MB-231

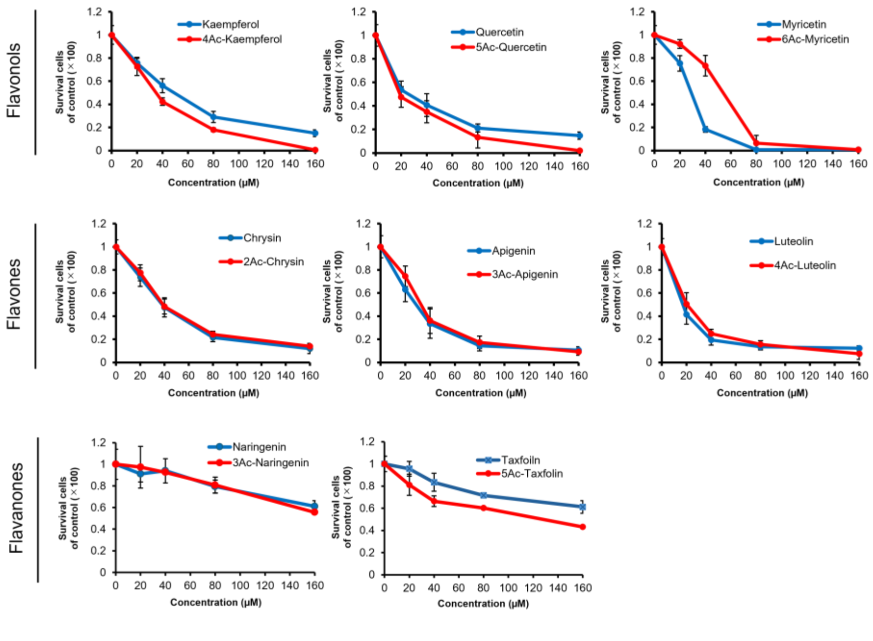

(b) HCT-116

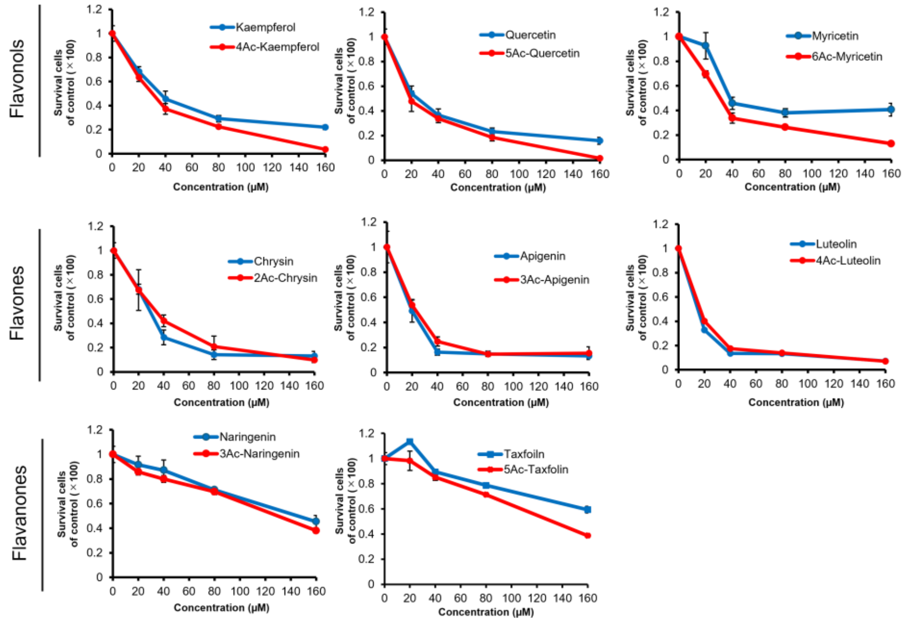

(c) HepG2

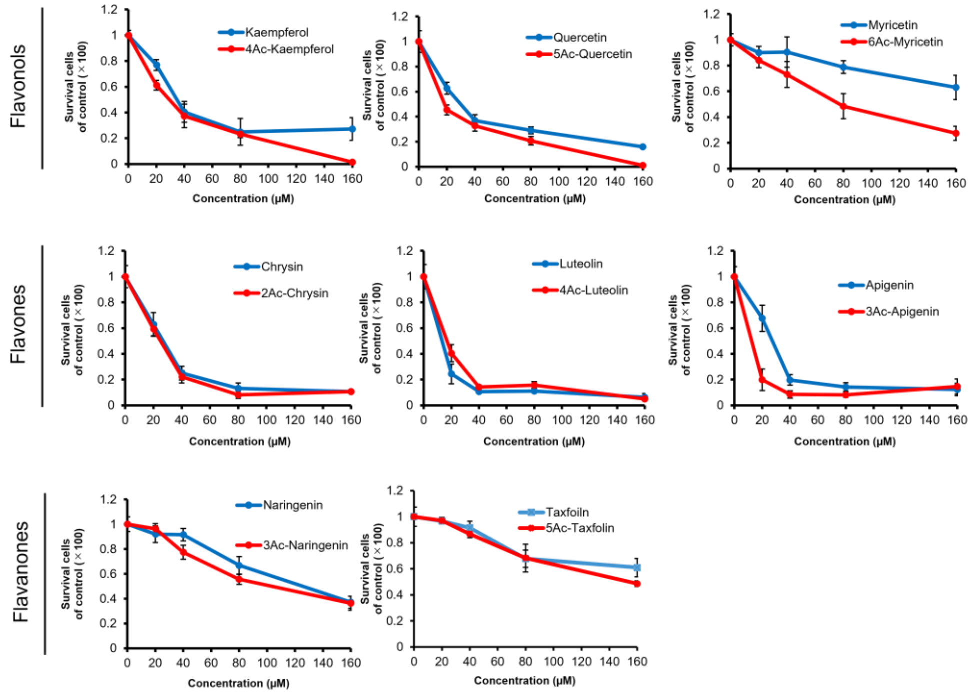

Figure S1. Effect of on the proliferation of (a) MDA-MB-231, (b) HCT-116 and (c) HepG2 cells. The cells were placed into 6-well plate and treated with indicated concentrations of each compounds or 0.1% DMSO (control) for 48 h. After cell collection, each cell solution was stained with 100 μL of 0.4% trypan blue solution for 1 minute and cells were counted using a hemocytometer.

The data shown represent the means ± SD of three or more independent experiments.

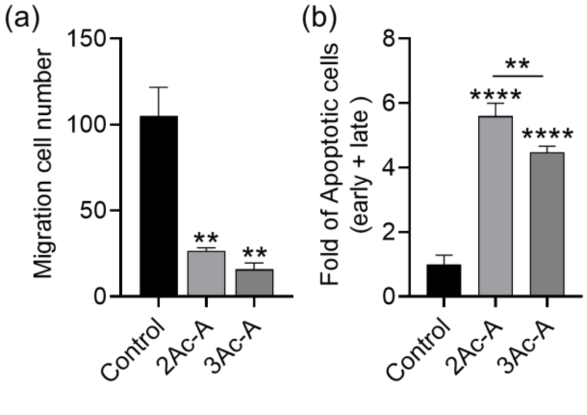

Figure S2. Differential effects of 2Ac-A and 3Ac-A on anti-migration and apoptosis induction. (a) The wound healing quantification of acetylated apigenin. The ability to inhibit cell migration was evaluated at the half of IC_{50} concentration of each compound. (b) Quantification of apoptosis rate (early apoptotic cells + late apoptotic cells) in MDA-MB-231 cells treated with IC_{50} of each of 2Ac-A and 3Ac-A for 48 h. The results shown (mean ± SD) are representative of two independent experiments (*n* = 3). **$p < 0.01$, ***$p < 0.001$, ****$p < 0.0001$: significant differences between control and each compound.
